# Supplementary material for: Eeyarestatin Compounds Selectively Enhance Sec61-Mediated Ca2+ Leakage from the Endoplasmic Reticulum
Source: Cell Chem Biol. 2019 Apr 18;26(4):571–583.e6. doi: 10.1016/j.chembiol.2019.01.010 (PMC6483976; doi:10.1016/j.chembiol.2019.01.010)
Supplement: Document S1. Figures S1–S5 [file mmc1.pdf]

Cell Chemical Biology, Volume *26*

Supplemental Information

Eeyarestatin Compounds Selectively Enhance Sec61-Mediated Ca^{2+} Leakage from the Endoplasmic Reticulum

Igor Gamayun, Sarah O'Keefe, Tillman Pick, Marie-Christine Klein, Duy Nguyen, Craig McKibbin, Michela Piacenti, Helen M. Williams, Sabine L. Flitsch, Roger C. Whitehead, Eileithyia Swanton, Volkhard Helms, Stephen High, Richard Zimmermann, and Adolfo Cavalié

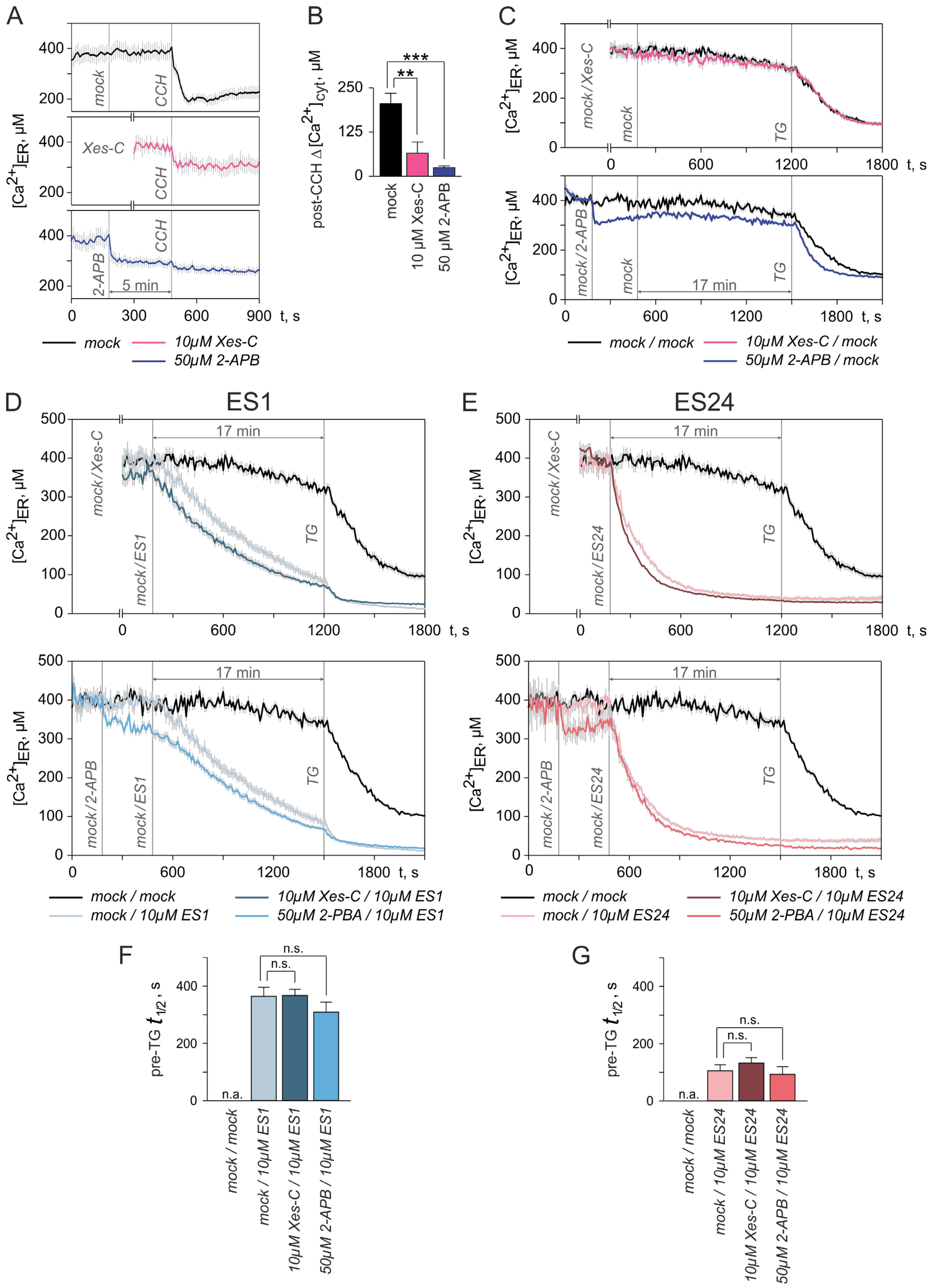

FIGURE S1 (related to Fig. 1)

FIGURE S1 (related to Fig. 1). Effects of ES1 and ES24 on the Ca^{2+} homeostasis under inhibition of IP_3 receptors.

(**A**) Assessing the inhibition of IP_3 receptors with 2-aminoethoxydiphenyl borate (2-APB) and xestospongin C (Xes-C) in HEK D1ER cells. The Ca^{2+} concentration in ER ($[Ca^{2+}]_{ER}$) was measured in the absence of extracellular Ca^{2+} (see Methods) and the functionality of the IP_3 signalling pathway was assessed by applying 1 mM carbachol (CCH). In mock-treated cells, CCH induced a drop in ER Ca^{2+}, indicating that the IP_3 signalling pathway is active in HEK D1ER cells (upper panel). The ER Ca^{2+} drop induced by CCH was strongly reduced in cells pre-treated with 10 μM Xes-C for 20 min (middle panel) and almost abolished after exposure to 50 μM 2-APB (lower panel).
(**B**) The quantification of decreases in $[Ca^{2+}]_{ER}$ induced by CCH (post-CCH $\Delta[Ca^{2+}]_{ER}$) indicates that 50 μM 2-APB inhibits IP_3 receptors stronger than 10 μM Xes-C in HEK D1ER cells.
(**C**) As a control, the protocol used in the experiments shown in Fig. 1B and Fig. 2C was tested in cells treated with Xes-C (10 μM Xes-C / mock) and 2-APB (50 μM 2-APB / mock). The time courses of $[Ca^{2+}]_{ER}$ in non-treated cells (mock / mock) and in cells treated with Xes-C were similar (upper panel). Although 2-APB induced a drop in ER Ca^{2+}, $[Ca^{2+}]_{ER}$ remained constant during the recordings (lower panel). As in Fig.1B and 2C, 1 μM thapsigargin (TG) was applied at the end of recordings.
(**D** – **E**) Effects of 10 μM ES1 and 10 μM ES24 on $[Ca^{2+}]_{ER}$ under inhibition of IP_3 receptors with Xes-C and 2-APB. HEK D1ER cells were pre-treated with 10 μM Xes-C for 20 min and, subsequently, ES1 and ES24 were applied (*upper panels*, 10 μM Xes-C / 10 μM ES1, 10 μM Xes-C / 10 μM ES24). The exposure to 2-APB was 5 min long and followed by application of ES1and ES24 (*lower panels*, 50 μM 2-APB / 10 μM ES1, 50 μM 2-APB / 10 μM ES24). For comparison, time courses of $[Ca^{2+}]_{ER}$ in control experiments (mock / mock) and the effects of ES1 and ES24 on naïve cells (mock / 10 μM ES1; mock / 10 μM ES24) are shown.
(**F** – **G**) Comparison of half-times of the $[Ca^{2+}]_{ER}$ decay induced by ES1 and ES24 before TG application (pre-TG $t_{1/2)}$ in the experiments shown in D-E.

DMSO controls are denoted as mock. Application time points are indicated by vertical lines in A and C-G. Data is presented as mean ± SEM. n.a., not analysed; n.s., non-significant.
**, $P < 0.01$; ***, $P < 0.001$. N: 9-15 cells per experimental setting.

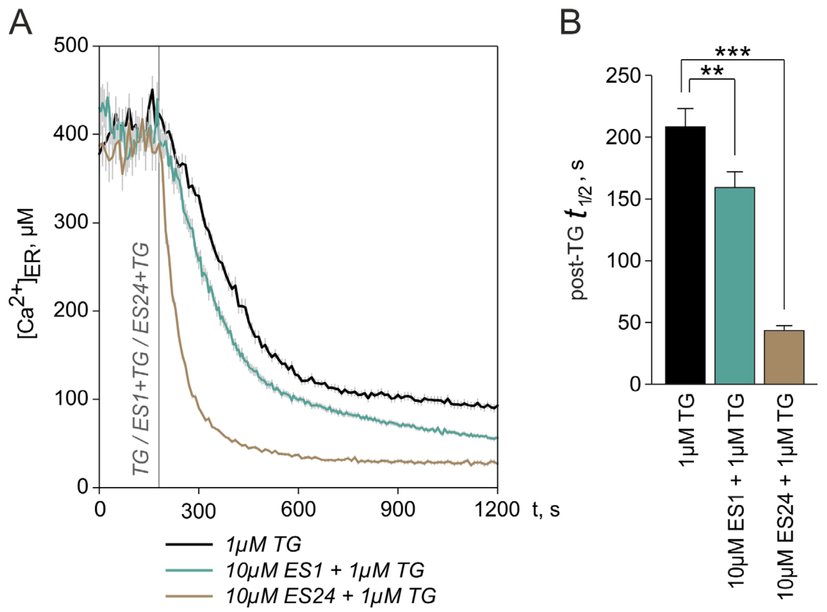

FIGURE S2 (related to Fig. 2). ES1 and ES24 accelerate the ER Ca^{2+} depletion induced by inhibition of SERCA pumps with thapsigargin.

(**A**) The Ca^{2+} concentration in ER ($[Ca^{2+}]_{ER}$) was measured in HEK D1ER cells in the absence of extracellular Ca^{2+} (see Methods) and 1 µM thapsigargin was applied either alone (TG) or in combination with 10 µM ES1 (TG+ES1) and 10 µM ES24 (TG+ES24) as indicated. (**B**) Comparison of the half times of decay in $[Ca^{2+}]_{ER}$ (post-TG $t_{1/2}$) induced by the application of TG, TG+ES1 and TG+ES24 in the experiments shown in A. Data is presented as mean ± SEM. **, $P < 0.01$; ***, $P < 0.001$. N: 10-13 cells per experimental setting.

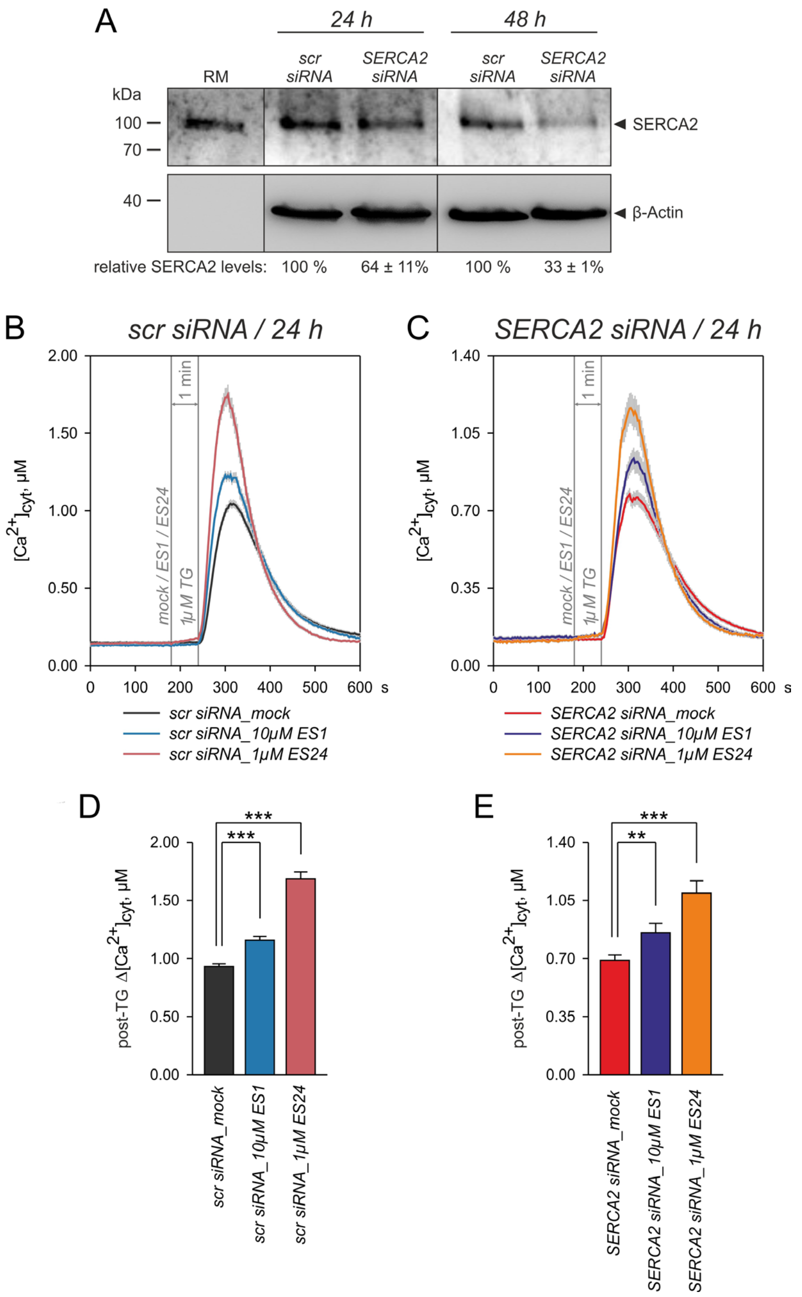

FIGURE S3 (related to Fig. 3). Effects of ES1 and ES24 in HeLa cells after knockdown of SERCA pumps.

(**A**) Expression of *SERCA2* in HeLa cells transfected with scramble siRNA (*scr siRNA*) or with siRNA targeting *SERCA2* (*SERCA2 siRNA*). SERCA2 protein levels were normalized to β-actin and expressed relative to levels in cells transfected with *scr siRNA*. RM, pancreatic rough microsome. Relative SERCA2 protein levels are given below Western blots.
(**B – C**) Effects of 10 µM ES1 and 1 µM ES24 on HeLa cells transfected with *scr siRNA* or *siRNA SERCA2*, as indicated above graphs.
(**D – E**) Quantification of TG responses (post-TG $\Delta[Ca^{2+}]_{cyt}$) measured in experiments shown in B-C.

DMSO controls are denoted as mock. Application time points of ES1, ES24 and TG are indicated by vertical lines in B-C. Data is presented as mean ± SD in A and as mean ± SEM in B-E. N: 3 blots (A) and 48-63 cells per experimental setting (B-H). **, P < 0.01 and ***, P < 0.001.

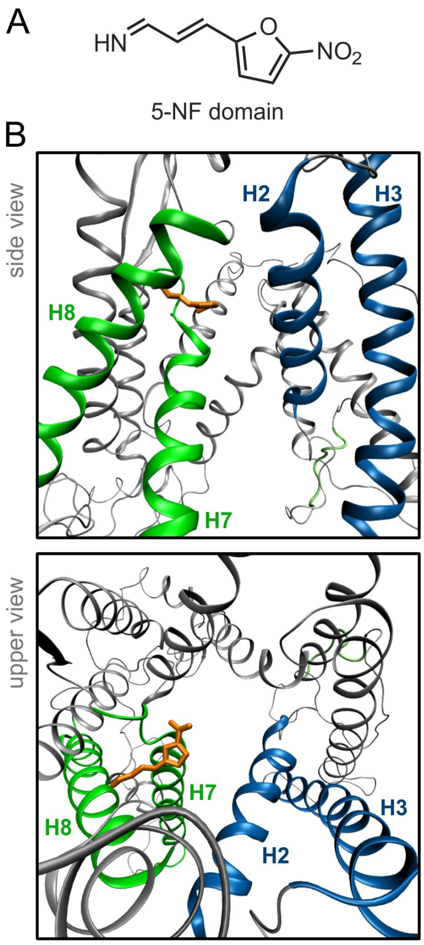

FIGURE S4 (related to Fig. 6). Docking of the 5-nitrofuran (5-NF) domain to Sec61α.

(**A**) Chemical structure of the 5-NF domain.
(**B**) Docking of the pure 5-NF domain was analysed using the homology model of human Sec61α. Shown is the docking position of the 5-NF domain (orange) with best docking score (ΔG = -5.69 kcal/mol). While this is lower than the docking scores of ES1 and ES24, it is typical that larger molecules achieve larger docking scores. The distance of 5-NF to the H2-H7 gap is 8.65 Å and, thus, slightly shorter than for ES1 (9.98 Å, see Fig. 6) and ES24 (9.87 Å, see Fig. 6). Images show the Sec61α model seen from the plane of the membrane with the cytosolic side upwards (upper panel) and from the cytosol (lower panel). Lateral gate helices are highlighted in colours (H2 and H3, blue; H7 and H8, green).

A

Mutation	Minimal distance (Å)
D60G	27.29
M65R	33.99
R66I	37.19
S71F	28.50
G80V	13.15
S82P	7.07
T86M	3.78
Y131H	18.27
M136T	26.43

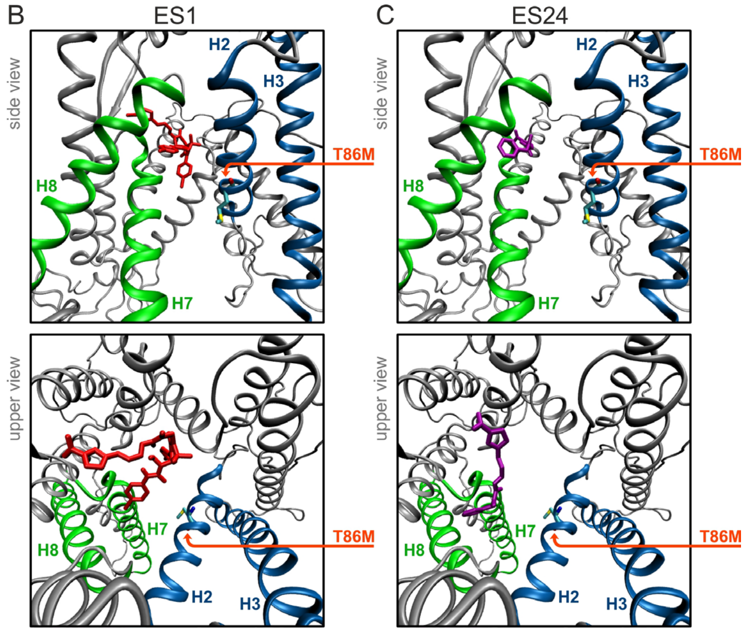

FIGURE S5 (related to Figure 6). Docking positions of ES1 and ES24 in the homology model of the human Sec61α with the mutation T86M.

(**A**) Previously described Sec61α mutations that confer resistance to various translocation inhibitors (Van Puyenbroeck and Vermiere, 2018) were incorporated in the homology model and minimal distances between mutated amino acids (C-alpha atom positions) and the H2-H7 gap of the lateral gate were measured. Among all these mutants, T86M is closest to the lateral gate. Hence, the mutation T86M was incorporated in the homology Sec61α model and docking of ES1 and ES24 to the structural model of this mutant was performed.
(**B** – **C**) Best-scored docking positions of ES1 and ES24 in the model of the T86M Sec61α mutant. The positions of ES1 and ES24 in the T86M Sec61α mutant are very similar to the respective docking positions in the model of native Sec61α (see Fig. 6). The docking scores of ES1 and ES24 in the T86M mutant were slightly lower (ES1, ΔG = -9.39 kcal/mol; ES24, ΔG = -8.57 kcal/mol) than in the native Sec61α (ES1, ΔG = -9.62 kcal/mol; ES24, ΔG = -9.04 kcal/mol; see Fig. 6). Images show side views (upper panels) and upper views (lower panels) of the Sec61α model seen from the plane of the membrane with the cytosolic side upwards and from the cytosol, respectively. The position of T86M is indicated in orange. Lateral gate helices are highlighted in colours (H2 and H3, blue; H7 and H8, green).
