# Supplementary material for: Eeyarestatin Compounds Selectively Enhance Sec61-Mediated Ca2+ Leakage from the Endoplasmic Reticulum
Source: Cell Chem Biol. 2019 Apr 18;26(4):571–583.e6. doi: 10.1016/j.chembiol.2019.01.010 (PMC6483976; doi:10.1016/j.chembiol.2019.01.010)
Supplement: Document S2. Article plus Supplemental Information [file mmc2.pdf]

Article

Cell Chemical Biology

Eeyarestatin Compounds Selectively Enhance Sec61-Mediated Ca^{2+} Leakage from the Endoplasmic Reticulum

Graphical Abstract

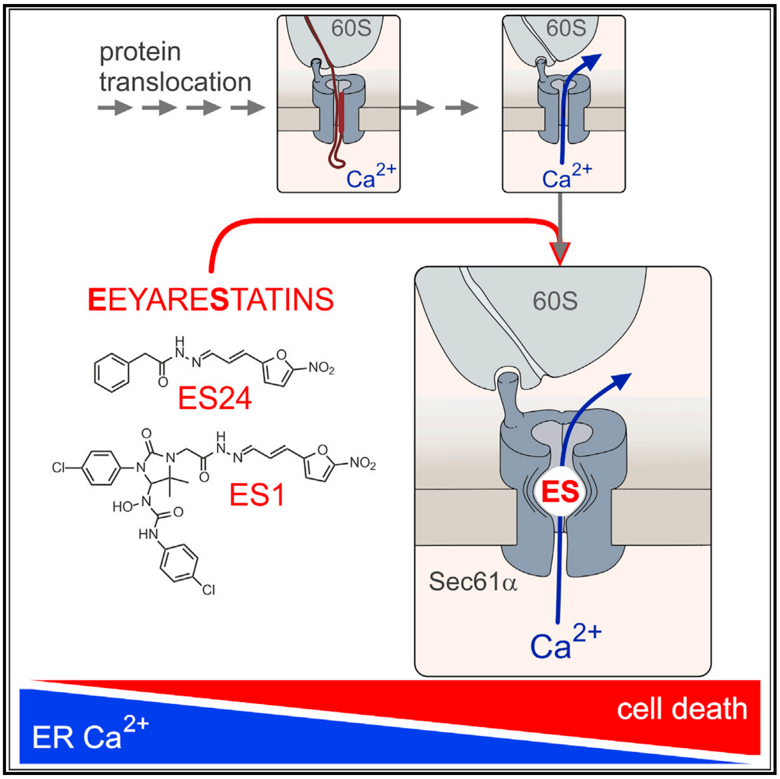

Highlights

- ES1, ES2, and ES24 deplete Ca^{2+} in ER
- ESR35 and ES47 do not affect cellular Ca^{2+} homeostasis
- The most potent eeyarestatin, ES24, comprises only the 5-nitrofuran domain
- ES1 and ES24 target Sec61 complexes in ER

Authors

Igor Gamayun, Sarah O'Keefe, Tillman Pick, ..., Stephen High, Richard Zimmermann, Adolfo Cavalié

Correspondence

igor.gamayun@uks.eu (I.G.), adolfo.cavalie@uks.eu (A.C.)

In Brief

Gamayun et al. discovered that eeyarestatins (ESs) interfere with closing mechanisms of Sec61 complexes of the ER and, as a "foot in the door," stabilize Sec61 complexes in a Ca^{2+}-permeable, open state. Specifically, ES24 enhances strongly the Sec61-mediated Ca^{2+} leakage from ER and induces Ca^{2+}-dependent cell death.

Gamayun et al., 2019, Cell Chemical Biology 26, 571–583
April 18, 2019 © 2019 The Authors. Published by Elsevier Ltd.
https://doi.org/10.1016/j.chembiol.2019.01.010

Cell Chemical Biology
Article

CellPress

Eeyarestatin Compounds Selectively Enhance Sec61-Mediated Ca^{2+} Leakage from the Endoplasmic Reticulum

Igor Gamayun,[1,*] Sarah O'Keefe,[2] Tillman Pick,[1] Marie-Christine Klein,[3] Duy Nguyen,[4] Craig McKibbin,[2,6] Michela Piacenti,[5,7] Helen M. Williams,[5,8] Sabine L. Flitsch,[5] Roger C. Whitehead,[5] Eileithyia Swanton,[2] Volkhard Helms,[4] Stephen High,[2] Richard Zimmermann,[3] and Adolfo Cavalié[1,9,*]
[1]Experimental and Clinical Pharmacology and Toxicology, Saarland University, 66421 Homburg, Germany
[2]School of Biological Sciences, Faculty of Biology, Medicine and Health, University of Manchester, Manchester M13 9PT, UK
[3]Medical Biochemistry and Molecular Biology, Saarland University, 66421 Homburg, Germany
[4]Center for Bioinformatics, Saarland University, 66123 Saarbrücken, Germany
[5]School of Chemistry, University of Manchester, Manchester M13 9PL, UK
[6]Present address: Royal Surrey County Hospital, Egerton Road, Guildford GU2 7XX, UK
[7]Present address: Eli Lilly Italia S.p.A., 50019 Sesto Fiorentino, Italy
[8]Present address: Unilever Research and Development, Port Sunlight, Bebington, Wirral CH63 3JW, UK
[9]Lead Contact
*Correspondence: igor.gamayun@uks.eu (I.G.), adolfo.cavalie@uks.eu (A.C.)
https://doi.org/10.1016/j.chembiol.2019.01.010

SUMMARY

Eeyarestatin 1 (ES1) inhibits p97-dependent protein degradation, Sec61-dependent protein translocation into the endoplasmic reticulum (ER), and vesicular transport within the endomembrane system. Here, we show that ES1 impairs Ca^{2+} homeostasis by enhancing the Ca^{2+} leakage from mammalian ER. A comparison of various ES1 analogs suggested that the 5-nitrofuran (5-NF) ring of ES1 is crucial for this effect. Accordingly, the analog ES24, which conserves the 5-NF domain of ES1, selectively inhibited protein translocation into the ER, displayed the highest potency on ER Ca^{2+} leakage of ES1 analogs studied and induced Ca^{2+}-dependent cell death. Using small interfering RNA-mediated knockdown of Sec61α, we identified Sec61 complexes as the targets that mediate the gain of Ca^{2+} leakage induced by ES1 and ES24. By interacting with the lateral gate of Sec61α, ES1 and ES24 likely capture Sec61 complexes in a Ca^{2+}-permeable, open state, in which Sec61 complexes allow Ca^{2+} leakage but are translocation incompetent.

INTRODUCTION

The endoplasmic reticulum (ER) is the intracellular organelle in which most secretory and many transmembrane proteins are folded and assembled in eukaryotic cells (Cross et al., 2009b; Lang et al., 2017; Rapoport et al., 2017). Early studies revealed that this organelle also stores Ca^{2+} in an ATP-dependent manner (Carreras-Sureda et al., 2018). It is now widely accepted that the ER represents the major intracellular Ca^{2+} reservoir and, thus, it is essential for both protein biogenesis and intracellular Ca^{2+} signaling in the mammalian cell. Generally, the total Ca^{2+} stored in ER are in the order of 1–3 mM and the luminal free Ca^{2+} concentration in ER ($[Ca^{2+}]_{ER}$) can reach 100–800 μM, which represents more than 1,000-fold of the cytosolic Ca^{2+} concentration ($[Ca^{2+}]_{cyt}$) at rest (Carreras-Sureda et al., 2018). However, the ER membrane is significantly more leaky than other cell membranes (Le Gall et al., 2004). Hence Ca^{2+}, among other cations, permeates the ER membrane and gives rise to Ca^{2+} leakage from ER as a result of the steep ER-cytosol Ca^{2+} gradient (Camello et al., 2002). Since ER Ca^{2+} leakage can be unmasked by inhibiting sarco/ER Ca^{2+} ATPases (SERCA) in multiple cell types, it is generally assumed that $[Ca^{2+}]_{ER}$ is maintained by balancing ER Ca^{2+} leakage and ATP-dependent Ca^{2+} influx into the ER via SERCA pumps (Camello et al., 2002; Carreras-Sureda et al., 2018). The molecular structure of Ca^{2+} leak channels in the ER has not yet been fully elucidated (Camello et al., 2002), but it is remarkable that the ubiquitously expressed Sec61 complex has been identified as a high-conductance Ca^{2+}-permeable channel that can support Ca^{2+} leakage from the ER (Simon and Blobel, 1991; Lomax et al., 2002; Wirth et al., 2003; Van Coppenolle et al., 2004; Erdmann et al., 2011). The core of the Sec61 complex consists of Sec61α, Sec61β, and Sec61γ, where Sec61α forms a protein-conducting channel that facilitates the translocation of secretory and transmembrane proteins into the ER (Lang et al., 2017). Apparently, Sec61 complexes become Ca^{2+} permeable only after protein translocation and the resulting Sec61-mediated Ca^{2+} leakage is limited by cytosolic Ca^{2+}-calmodulin and the ER luminal chaperone immunoglobulin heavy-chain binding protein under physiological conditions (Erdmann et al., 2011; Schäuble et al., 2012). Given the ubiquitous expression of Sec61 complexes in eukaryotic cells, Sec61-mediated Ca^{2+} leakage is emerging as an important pharmacological target within the field of Ca^{2+} homeostasis. However, little is known about small molecules that modulate ER Ca^{2+} leakage by targeting Sec61 complexes. For instance,

This is an open access article under the CC BY license (http://creativecommons.org/licenses/by/4.0/).

puromycin enhances the Sec61-mediated Ca^{2+} leakage (Lomax et al., 2002; Van Coppenolle et al., 2004; Lang et al., 2011a), but, mechanistically, puromycin is a substrate for the ribosomal peptidyltransferase and clears nascent polypeptide chains from protein-conducting channels in the ER membrane (Simon and Blobel, 1991). Some inhibitors of protein translocation across the ER membrane bind directly to the Sec61 complex and stabilize it in a closed state (van Puyenbroeck and Vermeire, 2018). With the exception of exotoxin A (Schäuble et al., 2014), however, it is not known whether Sec61 complexes that have been blocked by translocation inhibitors become permeable or are sealed for Ca^{2+} fluxes.

Eeyarestatin 1 and eeyarestatin 2 (ES1 and ES2) are small molecules that were initially identified as inhibitors of protein dislocation from ER into the cytosol (Fiebiger et al., 2004). Subsequent studies have shown that ES1 is an inhibitor of ER-associated protein degradation (ERAD), which targets the cytosolic AAA-ATPase p97, leading to the accumulation of poly-ubiquitinated ERAD substrates (Wang et al., 2008, 2010). Supporting this idea, it has been shown that ES1 is particularly cytotoxic against cancer cells when combined with proteasome inhibitors (Wang et al., 2009; Auner et al., 2013). Additional mechanisms that potentially contribute to the cytotoxic effects of ES1 are the impairment of both intracellular vesicular transport and membrane insertion of tail-anchored proteins (Aletrari et al., 2011; Naydenov et al., 2012; Figueiredo Costa et al., 2018). Independently of targeting the ER-cytosol dislocation of proteins, ES1 also inhibits the co-translational translocation of nascent polypeptides into the ER, most likely by inhibiting Sec61 complexes both *in cellula* and *in vitro* (Cross et al., 2009a). The ES1-dependent inhibition of protein translocation at the ER leads to an almost complete loss of glycoprotein secretion (Cross et al., 2009a) and, after ~2 h of exposure to ES1, cells also begin to show signs of ER stress in the form of the unfolded protein response (McKibbin et al., 2012). Thus, ES1 apparently affects ER functions at various levels, including co-translational protein translocation through Sec61 complexes (Cross et al., 2009a) and, since Sec61 complexes support Ca^{2+} fluxes through ER membranes (Lang et al., 2017), it can be predicted that ES1 treatment of cells will also impact on ER Ca^{2+} homeostasis. However, whether the ES1-mediated blockade of co-translational translocation is accompanied by the opening or closing of Sec61 complexes to Ca^{2+} ions is unknown. To distinguish between these two possibilities, we monitored the effects of ES1 by imaging both $[Ca^{2+}]_{cyt}$ and $[Ca^{2+}]_{ER}$. Surprisingly, we found that ES1 enhanced ER Ca^{2+} leakage and depleted the ER Ca^{2+} store. Using *SEC61A1* gene silencing, we show that ES1 increases Ca^{2+} leakage from ER by targeting Sec61 complexes. By comparing the actions of different ES1 analogs on both protein translocation and Ca^{2+} homeostasis, we pinpointed the structural domain responsible for the effects of ES1 on Sec61 complexes. Hence, ES24, which resembles the 5-NF domain of ES1 and blocks protein translocation *in vitro*, is more effective than the parent compound at enhancing Sec61-mediated Ca^{2+} leakage and is particularly cytotoxic to cells that are sensitive to Ca^{2+}-induced cell death. Conversely, the effects of ES24 on cellular ubiquitin homeostasis, and hence its likely impact on protein degradation, are less pronounced than those of ES1. On the basis of these findings, we propose that ES24 and the 5-NF domain of ES1 interact with the lateral gate as a "foot in the door" and capture Sec61 channels in a Ca^{2+}-permeable, open state that is translocation incompetent.

RESULTS

ES1 Depletes ER Ca^{2+} by Enhancing Ca^{2+} Leakage from ER

To obtain an integrated view of the Ca^{2+} status in cells under treatment with ES1, we measured free Ca^{2+} concentrations simultaneously in the ER ($[Ca^{2+}]_{ER}$) and the cytosol ($[Ca^{2+}]_{cyt}$) in the absence of extracellular Ca^{2+} to minimize Ca^{2+} entry. $[Ca^{2+}]_{ER}$ was imaged with the genetically encoded, FRET-based Ca^{2+} sensor D1ER (Palmer et al., 2004), which was stably expressed in HEK cells (HEK-D1ER). FURA-2 was used to image $[Ca^{2+}]_{cyt}$ in the same HEK D1ER cells. To minimize possible secondary effects, such as those resulting from a prolonged ER stress (see McKibbin et al., 2012), cellular Ca^{2+} levels were analyzed within minutes of ES1 addition in the so-called "online" protocol (Figure 1). In this approach, we first explored the action of ES1 on $[Ca^{2+}]_{cyt}$ and $[Ca^{2+}]_{ER}$. Afterward, we unmasked Ca^{2+} leakage from the ER by blocking SERCA pumps with thapsigargin (TG) and measured the specific effects of ES1 on Ca^{2+} leakage from the ER.

As shown in Figure 1A, we observed an increase of $[Ca^{2+}]_{cyt}$ immediately after application of ES1. Since we minimized extracellular Ca^{2+} entry by using EGTA in the bath solution, the increase of $[Ca^{2+}]_{cyt}$ induced by ES1 could in principle have resulted from the outflow of Ca^{2+} from intracellular Ca^{2+} stores. It is conceivable that ES1 potentiated the accumulation of ER Ca^{2+} in cytosol by also inhibiting cellular clearance mechanisms. However, all cells treated with 0.1 and 1 μM ES1 successfully removed the large amounts of Ca^{2+} that were released by TG into the cytosol (Figure 1A). Based on these observations, we suggest that ES1 does not disturb the clearance of Ca^{2+} from the cytosol, at least during the 17-min exposure to the compound. In addition, the fact that TG was largely ineffective after treatment with 10 μM ES1 (Figure 1A) underpinned the suggestion that ES1 primarily mobilizes Ca^{2+} from TG-sensitive Ca^{2+} stores. This suggestion is further supported by the analysis of $[Ca^{2+}]_{ER}$ that was measured simultaneously in the same set of cells (Figure 1B). The loss of ER Ca^{2+} due to EGTA in the bath solution was relatively low before TG application (~0.75% per min). By contrast, $[Ca^{2+}]_{ER}$ began to decrease immediately after application of 1–10 μM ES1 (Figure 1B). Thus, the $[Ca^{2+}]_{ER}$ imaging experiments strongly suggest that ES1 specifically impaired ER Ca^{2+} homeostasis in a dose-dependent manner. The possible involvement of IP_3 receptors in the effects of ES1 is unlikely because the decrease of $[Ca^{2+}]_{ER}$ was also observed in the presence of IP_3 inhibitors (Figure S1). One possible explanation for this effect is that ES1 might have inhibited SERCA pumps. A second, not necessarily exclusive, explanation is that ES1 might have enhanced Ca^{2+} leakage from the ER. We therefore inhibited SERCA pumps with TG in order to determine the specific effects of ES1 on Ca^{2+} leakage and found that ES1 apparently enhanced the Ca^{2+} leakage rate such that the TG-induced decay of $[Ca^{2+}]_{ER}$ became faster as the ES1 concentration was increased from 0.1 to 1 μM, suggesting that ES1 induced a dose-dependent gain of Ca^{2+} leakage (Figure 1B).

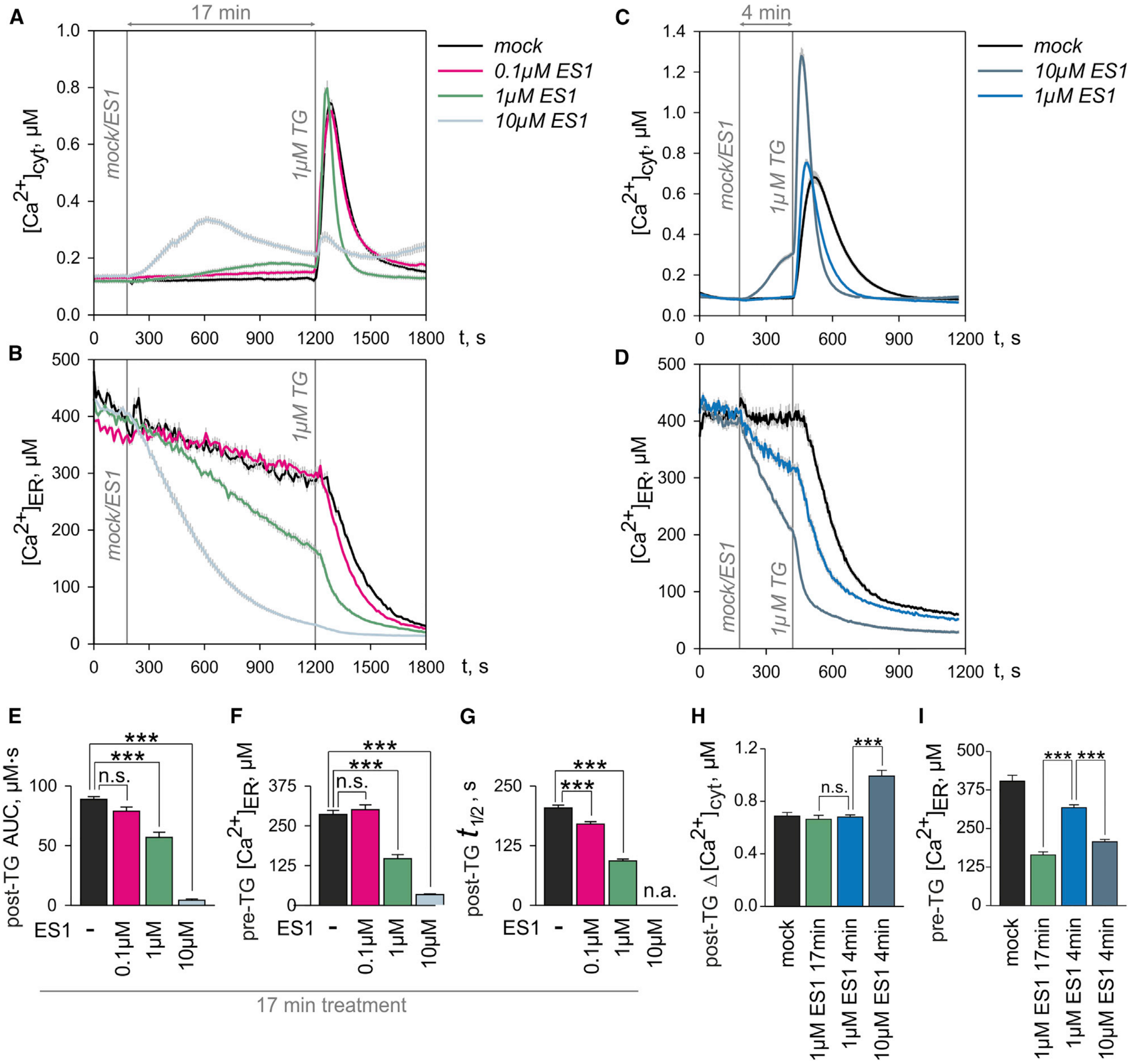

Figure 1. Eeyarestatin 1 (ES1) Interferes with the Cell Ca^{2+} Homeostasis by Enhancing the Ca^{2+} Leakage from ER in a Dose- and Time-Dependent Manner

(A and B) Increasing concentrations of ES1 produced accumulation of Ca^{2+} in cytosol (A), which resulted from the loss of ER (B) Ca^{2+} due to the dose-dependent gain of Ca^{2+} leakage.

(C and D) The TG responses in cytosolic Ca^{2+} (C) after short exposures to ES1 reflected the dose-dependent decrease of ER Ca^{2+} (D) and the gain of Ca^{2+} leakage.

(E) Surges in $[Ca^{2+}]_{cyt}$ after TG application were integrated between 1,200 and 1,800 s to obtain the area under the curve (post-TG AUC) for the experiments shown in (A).

(F) Decreases of $[Ca^{2+}]_{ER}$ induced by ES1 in experiments shown in (B) were quantified just before TG application (pre-TG $[Ca^{2+}]_{ER}$).

(G) The Ca^{2+} leakage from ER was estimated by measuring the half-time of the decay in $[Ca^{2+}]_{ER}$ (post-TG $t_{1/2}$) induced by TG in experiments shown in (B).

(H and I) The relative amplitude of $[Ca^{2+}]_{cyt}$ increases after TG applications (post-TG $\Delta[Ca^{2+}]_{cyt}$) (H) and $[Ca^{2+}]_{ER}$ levels just before TG application (pre-TG $[Ca^{2+}]_{ER}$) (I) are shown for the experiments in (C and D).

DMSO controls are denoted as mock. Application time points are indicated by vertical lines in (A–D) (mock/ES1; 1μM TG). Data are presented as means ± SEM. n.a., not analyzed; n.s., not significant; ***$p < 0.001$. N = 30–47 cells per experimental setting. See also Figure S1.

When the ER Ca^{2+}-efflux-influx equilibrium was broken down by TG, the $[Ca^{2+}]_{ER}$ decay displayed a typical exponential time course (Figure 1B), as reported previously (e.g., Means et al., 2006). Accordingly, the amount of Ca^{2+} running out per unit time, here termed Ca^{2+} leakage, is proportional to the product of $[Ca^{2+}]_{ER}$ and the Ca^{2+} leak constant, which in turn reflects

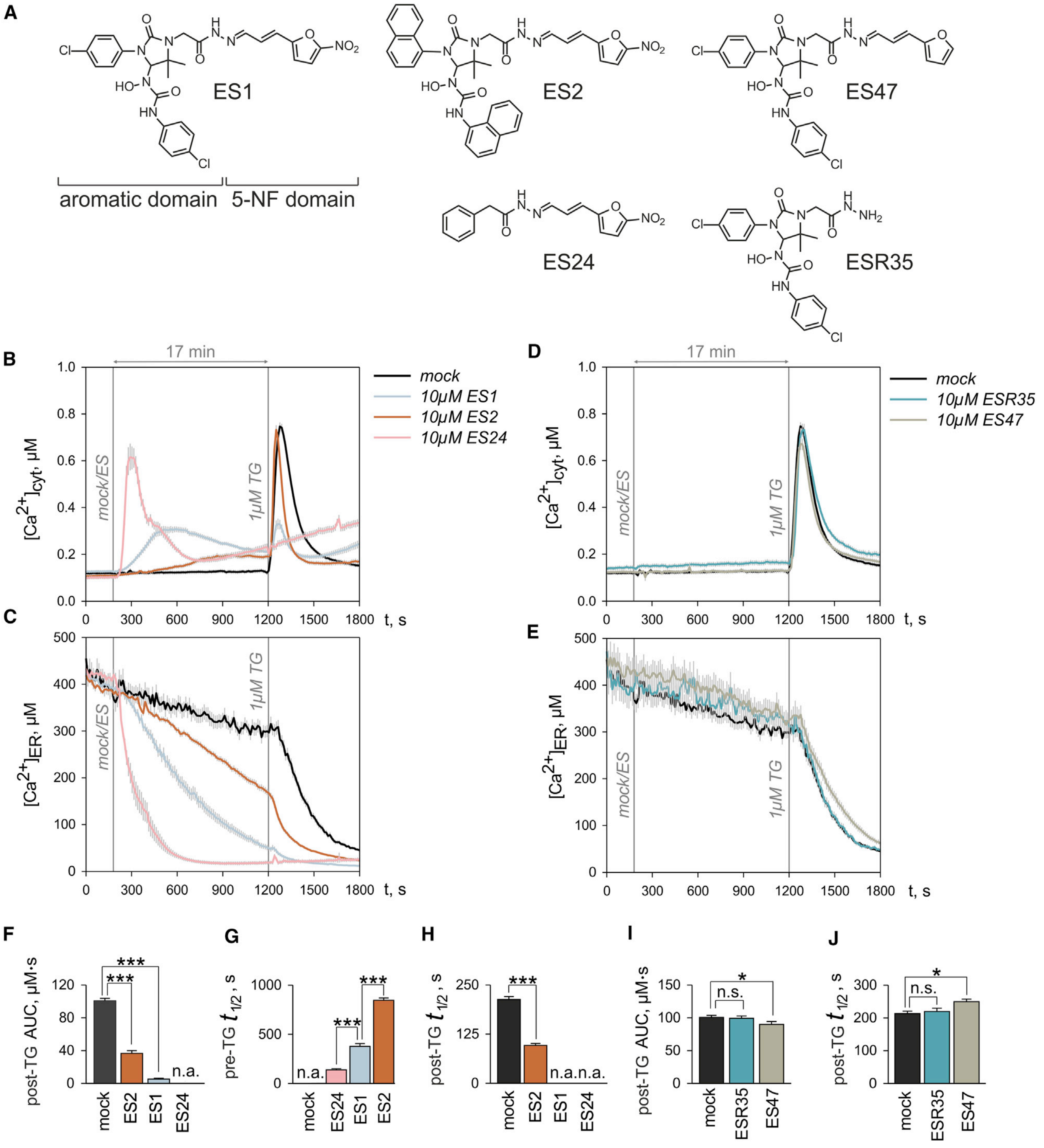

Figure 2. Comparison of the Effects of ES1 and Its Analogs ES2, ES24, ESR35, and ES47 on Cell Ca^{2+} Homeostasis

(A) ES1 comprises an aromatic-rich domain and a domain that contains a 5-nitrofuran (5-NF) group. The ES1 analogs carry chemical modifications either in the aromatic domain (ES2, ES24) or in the 5-NF domain (ESR35, ES47).

(B and C) The effects of ES2 on cytosolic (B) and ER (C) Ca^{2+} were mild compared with those of ES1, while ES24 rapidly depleted ER Ca^{2+} producing a robust accumulation of Ca^{2+} in cytosol.

(D and E) ESR35 was ineffective at all and ES47 produced minute effects on cytosolic (D) and ER (E) Ca^{2+}.

(F) The area under the curve for TG responses shown in (A) were measured by integrating time courses of $[Ca^{2+}]_{cyt}$ between 1,200 and 1,800 s (post-TG AUC).

(G and H) Half-times were determined for $[Ca^{2+}]_{ER}$ decays (G) after application of ES1, ES2, and ES24 (pre-TG $t_{1/2}$) and (H) after the subsequent TG application in experiments shown in (C) (post-TG $t_{1/2}$).

(legend continued on next page)

the fraction of $[Ca^{2+}]_{ER}$ released per unit time and is directly related to half-time of decay ($t_{1/2}$), i.e., the time required for $[Ca^{2+}]_{ER}$ to decrease by 50% of the pre-TG level. Since the TG-induced changes in $[Ca^{2+}]_{cyt}$ likely reflect the Ca^{2+} leakage, it is expected that a time-dependent decrease of $[Ca^{2+}]_{ER}$ due to enhanced Ca^{2+} leakage will paradoxically reduce cytosolic responses to TG, as is observed for 10 μM ES1 in Figure 1A. Therefore, we next analyzed the time-dependent effects of ES1 on $[Ca^{2+}]_{cyt}$ and $[Ca^{2+}]_{ER}$. Despite the lower $[Ca^{2+}]_{ER}$ levels, the cytosolic responses to TG were higher in cells treated with 10 μM ES1 for 4 min than in those treated with 1 μM ES1 (Figure 1C). In 4-min treatments, however, the $[Ca^{2+}]_{ER}$ levels decayed to lower levels in cells exposed to 10 μM ES1 than in those treated with 1 μM ES1 (Figure 1D). The most likely explanation for these apparently contradictory results is that 10 μM ES1 enhanced the Ca^{2+} leakage sufficiently strongly to compensate for the reduced $[Ca^{2+}]_{ER}$ levels, thus giving rise to stronger cytosolic responses to TG. In fact, $t_{1/2}$ of the TG-induced decay of $[Ca^{2+}]_{ER}$ was 106.61 ± 7.72 and 43.83 ± 2.18 s in cells treated for 4 min with 1 and 10 μM ES1, respectively. Next, we compared the effects of 4- and 17-min exposure with 1 μM ES1. Since $[Ca^{2+}]_{ER}$ decreased continuously during ES1 exposures, the $[Ca^{2+}]_{ER}$ levels were significantly lower after a 17-min exposure than after a 4-min exposure to 1 μM ES1 (Figures 1B and 1D). Surprisingly, however, the cytosolic responses to TG were similar after 4- and 17-min treatments with 1 μM ES1 (Figures 1A and 1C). Based on the idea that the product of $[Ca^{2+}]_{ER}$ and Ca^{2+} leakage constant determines the amplitude of cytosolic responses to TG, the most likely explanation for the different responses at 4 and 17 min is that the gain of Ca^{2+} leakage was high enough to support a robust response to TG in cytosol at the end of the 17-min ES1 treatment. In fact, $t_{1/2}$ of the TG-induced decay of $[Ca^{2+}]_{ER}$ was 106.61 ± 7.72 and 94.20 ± 6.50 s after 4- and 17-min treatments with 1 μM ES1, respectively. Thus, the experiments in Figures 1C and 1D indicate that ES1 begins to enhance the Ca^{2+} leakage from ER almost immediately after application and this effect is boosted during the treatment in a time-dependent manner.

A quantitative analysis of the Ca^{2+} imaging data revealed the dose-dependence of ES1 effects at the level of cytosolic Ca^{2+} and ER Ca^{2+}. The area under the $[Ca^{2+}]_{cyt}$ surge induced by TG (post-TG area under the curve [AUC]), the $[Ca^{2+}]_{ER}$ levels before TG application (pre-TG $[Ca^{2+}]_{ER}$) and the half-times of the TG-induced decay in $[Ca^{2+}]_{ER}$ (post-TG $t_{1/2}$) exhibited a strong dependence on ES1 concentrations in the 17-min treatments (Figures 1E–1G). Specifically, post-TG $t_{1/2}$ showed the dose-dependent gain of Ca^{2+} leakage induced by ES1 (Figure 1G). Based on the analysis of post-TG AUC, pre-TG $[Ca^{2+}]_{ER}$ and post-TG $t_{1/2}$, it can be estimated that the half maximal effective concentration (EC_{50}) for the effects of ES1 on cytosolic and ER Ca^{2+} is approximately 1 μM (Figures 1E–1G). The low post-TG AUC values in the presence of 1 μM ES1 (Figure 1E) reflected mainly the shortening of the whole TG response (Figure 1A), which in turn paralleled the decrease of $[Ca^{2+}]_{ER}$ (Figure 1F) and the increase of the Ca^{2+} leak constant, seen as a shortening of post-TG $t_{1/2}$ (Figure 1G). Thus, ES1 affects the product of $[Ca^{2+}]_{ER}$ and Ca^{2+} leak constant, i.e., the Ca^{2+} leakage. Following this reasoning, the time-dependent ES1 effects on Ca^{2+} leakage can be appreciated by comparing 4- and 17-min treatments. Cytosolic TG responses displayed similar amplitudes (post-TG $\Delta[Ca^{2+}]_{cyt}$) after 4- and 17-min treatments with 1 μM ES1 (Figure 1H) despite lower $[Ca^{2+}]_{ER}$ levels after 17-min exposures (Figure 1I) because the Ca^{2+} leak constant increased with time, i.e., post-TG $t_{1/2}$ values decreased (106.61 ± 7.72 versus 94.20 ± 6.50 s for 4- and 17-min treatments with 1 μM ES1, respectively).

Differential Effect of ES1 Analogs on ER Ca^{2+}

ES1 comprises two structural and functional distinct domains (Figure 2A): a 5-NF-containing domain and an aromatic domain (Wang et al., 2010; Aletrari et al., 2011; McKibbin et al., 2012). In our present study, we have used the closely related analog ES2 and compounds ES24, ESR35, and ES47 (Figure 2A), collectively termed ESs which differ in either the aromatic or 5-NF domain (McKibbin et al., 2012).

ES1 enhanced Ca^{2+} leakage from the ER in a dose-dependent manner leading to a dose-dependent attenuation of the cytosolic responses to TG, seen as a decrease of post-TG AUC values (Figure 1). Using this paradigm for the action of ES1 on Ca^{2+} homeostasis and the online protocol, we analyzed the action of ES compounds at the highest concentration tested with ES1, namely 10 μM (Figure 2). The most obvious effects were seen immediately after application of ES1, ES2, and ES24 in the bath solution (Figures 2B and 2C). Strikingly, the increase of $[Ca^{2+}]_{cyt}$ was steeper and the decrease of $[Ca^{2+}]_{ER}$ faster upon treatment with ES24 than with either ES1 or ES2 (Figures 2B and 2C). Accordingly, the half-time of $[Ca^{2+}]_{ER}$ decay before TG application (pre-TG $t_{1/2}$) was approximately two times shorter for ES24 than for ES1 at 10 μM (Figure 2G). Therefore, the ER was completely empty at the end of the 17-min treatment with ES24 and cytosolic TG responses were absent (Figure 2F), meaning that we were not able to estimate the actual increase in Ca^{2+} leakage induced by ES24, i.e., post-TG $t_{1/2}$ (Figure 2H). As an alternative, we applied TG and ES24 simultaneously and found that this combination results in a 5-fold reduction in post-TG $t_{1/2}$, suggesting that ES24 is a potent enhancer of Ca^{2+} leakage (Figure S2). We also observed a continuous increase of $[Ca^{2+}]_{cyt}$ after 10–12 min exposure to ES24, pointing toward the risk of non-selective, cytotoxic effects of ES24 at prolonged exposures (Figure 2B). By contrast to ES24, the effects of ES2 on both $[Ca^{2+}]_{cyt}$ and $[Ca^{2+}]_{ER}$ were less pronounced than those of ES1 (Figures 2B and 2C). The pre-TG $t_{1/2}$ for the decay of $[Ca^{2+}]_{ER}$ induced by ES2 was about twice as long as in the presence of ES1 (Figure 2G). Accordingly, the loss of ER Ca^{2+} and the Ca^{2+} accumulation in cytosol were less pronounced under the treatment with ES2 (Figure 2C) and cytosolic TG responses were detectable (Figure 2F). In contrast, ESR35, which has previously been used as an inactive control

(I and J) The area under the curve of TG responses in the experiments shown in (D) (post-TG AUC) (I) and the half-time of TG-induced decay of $[Ca^{2+}]_{ER}$ shown in (E) (post-TG $t_{1/2}$) (J) were quantified for the time between 1,200 and 1,800 s.

Time points of ES and TG application are indicated with vertical lines in (A–D) (mock/ES; 1 μM TG). Mock denotes DMSO controls. Data are presented as means ± SEM. n.a., not analyzed; n.s., not significant; *p < 0.05; ***p < 0.001. N = 25–48 cells per experimental setting. See also Figure S2.

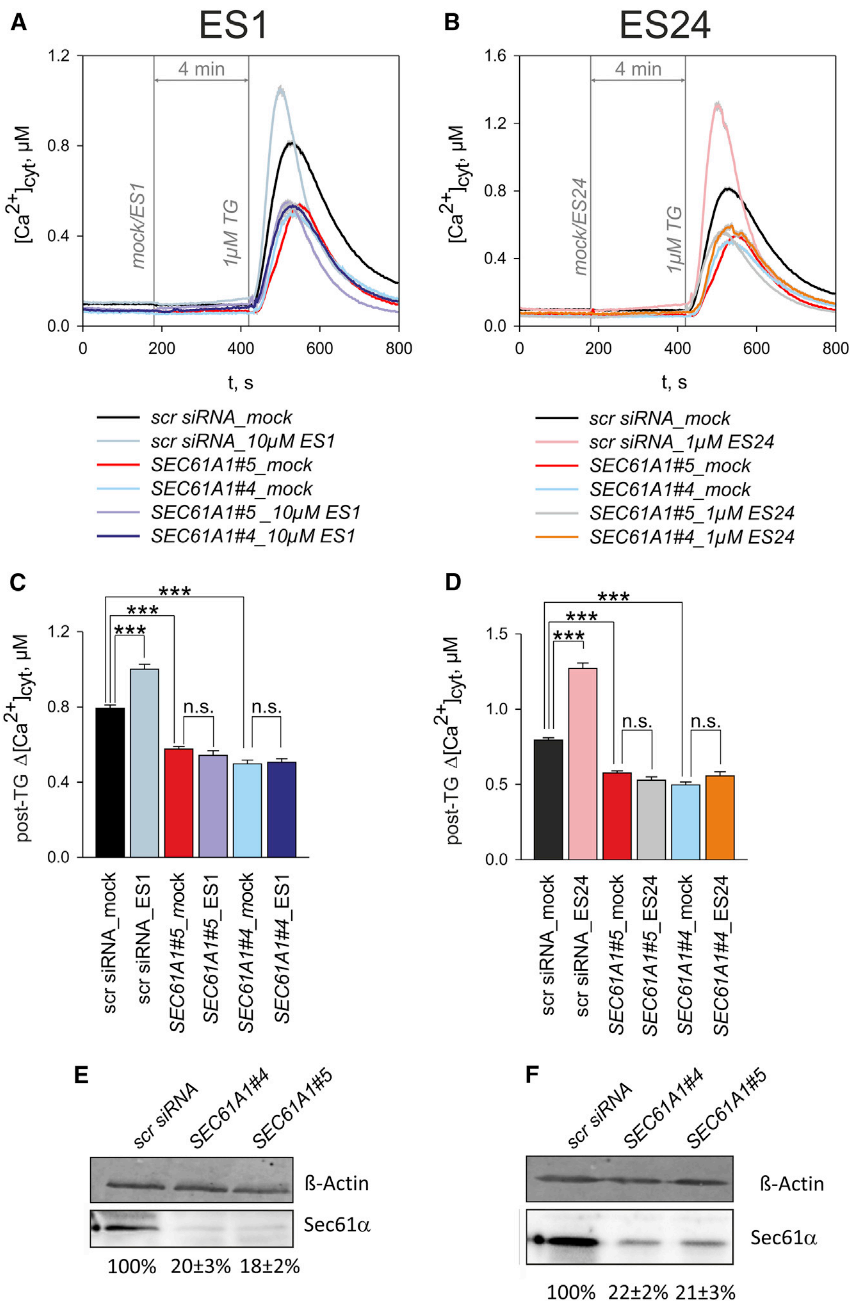

Figure 3. siRNA-Mediated Silencing of *SEC61A1* Abolished the Effects of ES1 and ES24 on Ca^{2+} Homeostasis

(A and B) The time courses of TG responses in cytosolic Ca^{2+} illustrate the loss of effects of ES1 (A) and ES24 (B) on Ca^{2+} leakage after silencing *SEC61A1* with two different siRNAs (*SEC61A1#4* and *SEC61A15*). Experiments with control siRNA (scrRNA) and DMSO controls (mock) are superimposed for comparison. Application times are indicated by vertical lines (mock/ES1, mock/ES24, 1 μM TG).

(C and D) Relative amplitudes of cytosolic TG responses (post-TG $\Delta[Ca^{2+}]_{cyt}$) were used to quantify the effects of ES1 (C) and ES24 (D) in the experiments illustrated in (A and B), respectively.

(E and F) Relative contents of Sec61α protein in cells treated with *SEC61A1* siRNAs are given as percentage of the Sec61α content in cells treated with *scrRNA* for the experiments with ES1 (E) and ES24 (F).

Data obtained in six different silencing experiments are presented as means ± SEM. n.s., not significant; ***$p < 0.001$. N = 94–222 cells per experimental setting. See also Figure S3.

(Cross et al., 2009a; McKibbin et al., 2012), was largely ineffective on both $[Ca^{2+}]_{cyt}$ and $[Ca^{2+}]_{ER}$ (Figures 2D and 2E). ES47, however, displayed a small but statistically significant inhibition of Ca^{2+} leakage from ER (Figure 2I) that was paralleled by an increase in the post-TG half-time by circa 16% (Figure 2J).

The 5-NF Domain of ES1 Enhances Ca^{2+} Fluxes through Sec61 Complexes

To examine the mechanism through which ES1 enhances Ca^{2+} leak, cells were depleted of Sec61α using small interfering RNA (siRNA). We performed these experiments in HeLa cells, in which silencing efficiencies of about 80% are routinely achieved (Lang et al., 2011b). Two independent siRNAs, *SEC61A1#4* and *SEC61A1#5*, were used to silence *SEC61A1*, and a scrambled siRNA (scr siRNA) was used as a control. Following our online protocol, HeLa cells were exposed to 10 μM ES1 and ES24, and the amplitude of cytosolic TG responses (post-TG $\Delta[Ca^{2+}]_{cyt}$) was measured as an indicator for Ca^{2+} leakage (Figures 3A and 3B). In cells treated with either of two independent siRNAs, *SEC61A1#4* and *SEC61A1#5*A, we consistently observed lower peaks in the cytosolic responses to TG (Figures 3A and 3B), and the post-TG $\Delta[Ca^{2+}]_{cyt}$ was about 35%–40% lower than in scr siRNA-treated cells (Figures 3C and 3D). Similar effects of *SEC61A1* silencing on cytosolic TG responses have been observed previously and were attributed to a decrease in Sec61-mediated Ca^{2+} leakage from ER (Erdmann et al., 2011). Western blot analysis confirmed that the two siRNAs reduced the Sec61α subunit by circa 80%–82% in HeLa cells (Figures 3E and 3F). The cytosolic TG responses of scr siRNA-treated HeLa cells exposed to ES1 and ES24 peaked higher and were of shorter duration than mock treated cells (Figures 3A and 3B), as for HEK D1ER cells (cf. Figures 1 and 2). Accordingly, post-TG $\Delta[Ca^{2+}]_{cyt}$ values were approximately 32% higher in the scr siRNA cells exposed to 10 μM ES1 (Figures 3 A and 3C). However, 10 μM ES1 failed to increase post-TG $\Delta[Ca^{2+}]_{cyt}$ in the cells treated with *SEC61A1#4* or *SEC61A1#5* (Figures 3 A and 3C). Similarly, 1 μM ES24 enhanced post-TG $\Delta[Ca^{2+}]_{cyt}$ values by about 62% in scr siRNA-treated cells but it was completely ineffective after silencing *SEC61A1* (Figures 3 B and 3D). A potential explanation

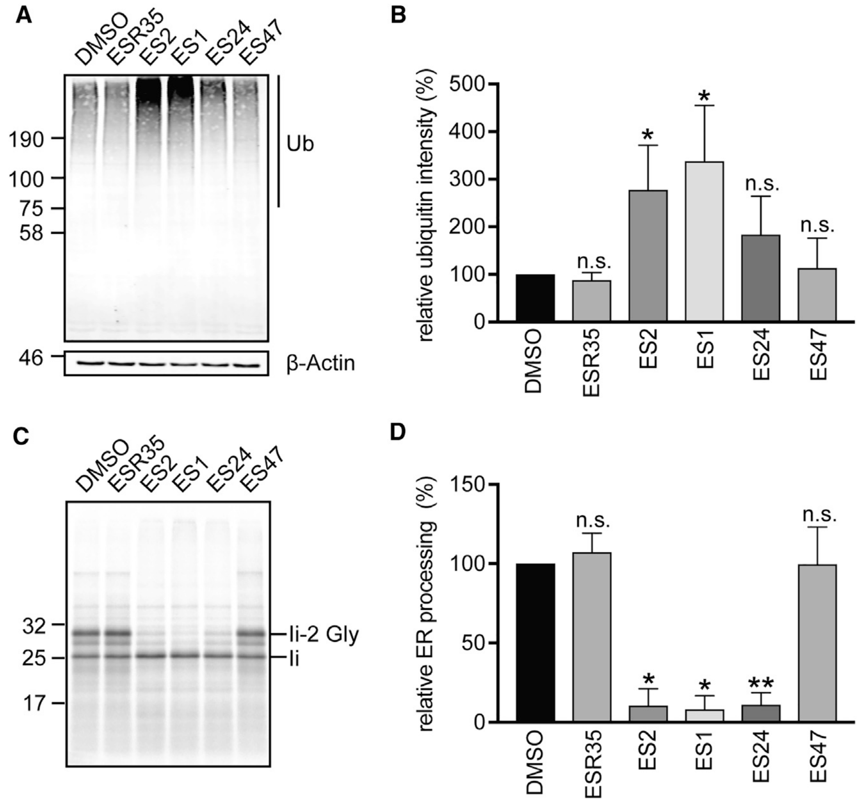

Figure 4. Differential Effects of ES1 Analogs on Protein Translocation and Degradation

(A and B) HeLa cells were treated with ES1 analogs (8 μM) or with equal amounts of DMSO for 8 h. Cell lysates were analyzed by immunoblotting with an anti-ubiquitin (Ub) antibody (A). Ubiquitin signals were normalized relative to β-actin loading controls and normalized ubiquitin intensities are expressed relative to DMSO controls (B). The total levels of poly-ubiquitinated proteins were markedly increased in cells treated with ES1 and ES2 but not with ESR35 and ES47, while the effects of ES24 were not statistically significant compared with DMSO controls.

(C and D) ES1, ES2, and ES24 reduced *in vitro* the glycosylation of a model membrane glycoprotein, the invariant chain (Ii) of the class II major histocompatibility complex, while ESR35 and ES47 had no inhibitory effect. Fully glycosylated Ii is denoted by Ii-2 Gly (C). Pancreatic ER ribosomes were preincubated for 1 h with ES1 analogs (250 μM) or with equal amounts of DMSO. Ratios of signal intensities Ii-2 Gly/Ii were used as a measure for total protein translocation and expressed relative to DMSO controls to estimate the relative ER processing (D). Quantifications are given as means ± SEM for three translocation experiments and six ubiquitination assays. n.s., not significant; *$p < 0.05$; **$p < 0.01$.

for this finding is that ES1 and ES24 were not effective after silencing *SEC61A1* because of low post-TG $\Delta[Ca^{2+}]_{cyt}$ levels. As an additional control, we have therefore reduced the amplitude of cytosolic TG responses via an independent mechanism, namely by reducing SERCA protein levels to about 60% using *siRNA SERCA2*. Under these conditions, the levels of post-TG $\Delta[Ca^{2+}]_{cyt}$ were as low as those measured after silencing *SEC61A1* and, remarkably, we found that 10 μM ES1 and 1 μM ES24 enhanced the cytosolic TG responses (Figure S3). Thus, these results strongly suggest that the mechanism by which ES1 and ES24 disrupt cellular Ca^{2+} homeostasis is via a specific enhancement of Sec61-mediated Ca^{2+} leakage. Hence, we conclude that ER resident Sec61 complexes are targets for both ES1 and ES24.

Differential Effects of ES Compounds on Protein Degradation and ER Translocation

ES1 interacts with the p97 complex and influences deubiquitinating processes that are mediated by p97-associated enzymes thereby inhibiting downstream proteasomal degradation and protein quality control (Wang et al., 2008). One of the hallmarks of this effect is an accumulation of high-molecular-weight poly-ubiquitinated species in cells exposed to ES1 for several hours (Wang et al., 2008; McKibbin et al., 2012), and we used this as a readout to compare the effects of our ES compounds on cellular ubiquitin homeostasis and related protein quality control processes. As described previously (Wang et al., 2008; McKibbin et al., 2012), the treatment of HeLa cells with 8 μM ES1 induced a ~3.4-fold increase of total poly-ubiquitinated species when compared with control cells (Figures 4A and 4B). Likewise, a substantial, accumulation of poly-ubiquitinated species was also observed in cells treated with ES2 (Figures 4A and 4B). In contrast, the effects of ES24 were by far less robust and, although a modest qualitative increase in signal was sometimes apparent (Figure 4A, cf. lanes 1 and 4), quantification showed that any changes were not statistically significant when compared with cells treated either with DMSO or ESR35 (Figure 4B). We speculate that the absence of any clear perturbation of ubiquitin homeostasis following treatment with ES24 reflects its lack of the distinct aromatic domain present in ES1 (cf. Figure 2A). This region is proposed to enhance the inhibitory effects of ES1 on the degradation of misfolded membrane proteins by targeting the compound to the ER where it may preferentially inhibit membrane-bound p97 (Wang et al., 2010). ES47 was also ineffective at causing any accumulation of poly-ubiquitinated species (Figure 4B) and, since it lacks an intact 5-NF domain (cf. Figure 2A), our findings support the proposal that this region is important for ES1-mediated inhibition of p97 (Wang et al., 2010). In summary, of the ES compounds that we analyzed, only ES1 and ES2 appear to cause the accumulation of endogenous poly-ubiquitinated species in HeLa cells, most likely via their inhibition of protein degradation (Fiebiger et al., 2004; Wang et al., 2010; McKibbin et al., 2012).

Since it has been shown that ES1 and ES2 inhibit Sec61-mediated protein translocation into the ER (Cross et al., 2009a), we studied the effects of our ES compounds using an -established cell-free membrane translocation assay. The histocompatibility leukocyte antigen (HLA) class II histocompatibility antigen gamma chain (Ii) was used as a model membrane glycoprotein, and its integration into ER-derived canine

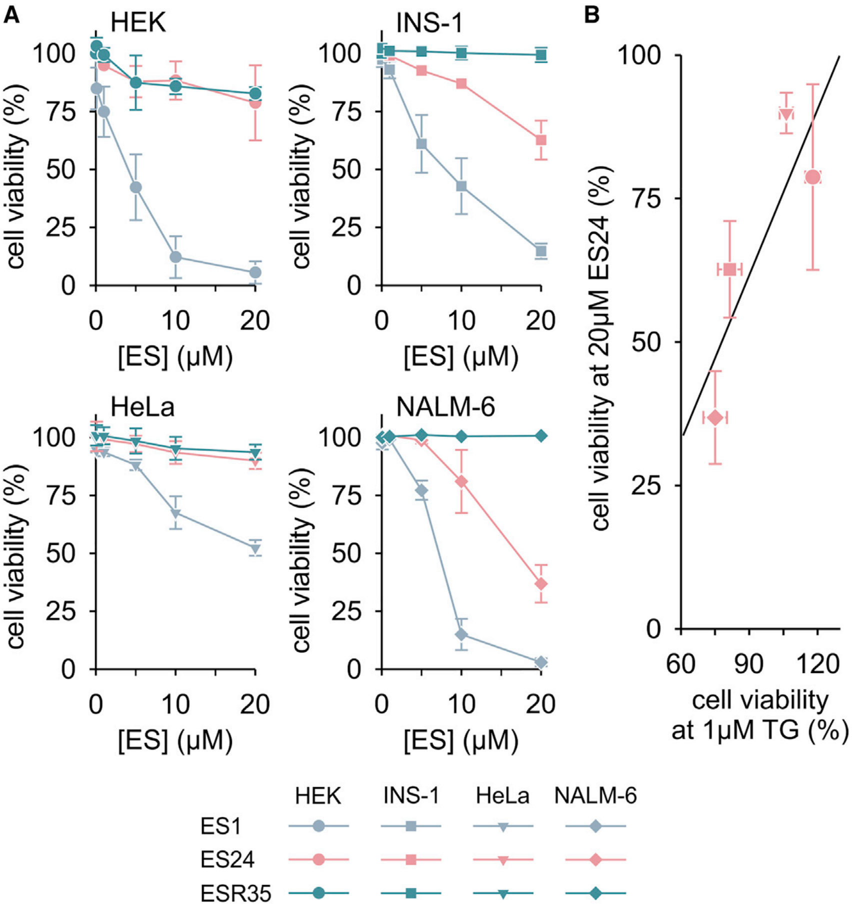

Figure 5. ES24 Reduces Cell Viability in a Ca^{2+}-Dependent Manner

(A) HEK, INS-1, HeLa, and NALM-6 cells were exposed for 24 h to various ES compounds (ES) and cell viability was measured as the percentage of living cells. While ES1 was cytotoxic to all cell lines, ESR35 was not cytotoxic at all and ES24 displayed mild effects on some cell lines.

(B) Comparison of cell viabilities for 24-h treatments with 20 μM ES24 and 1 μM TG. A strong Ca^{2+}-dependent component in cytotoxicity of ES24 is suggested by the linear correlation (black line) of the cell viabilities measured in the individual cell lines after 24 h treatment with 20 μM ES24 and 1 μM TG. Color and symbol coding for cell lines and substances are shown below panels. Cell viability was measured per duplicate in three independent experiments, normalized to DMSO controls and is presented as means ± SD.

pancreatic microsomes that had been pre-incubated with the different ES compounds was investigated as described previously (Cross et al., 2009a). Hence, the efficiency of the Sec61-mediated translocation of the N-terminal domain of Ii into the ER lumen was determined by quantifying its N-glycosylation. As reported previously, ES1 and ES2 inhibited membrane translocation by ~90% (Figures 4C and 4D), while ESR35 had no effect (Cross et al., 2009a). Strikingly, the effects of ES24 and ES47 on protein translocation mirrored their effects on Sec61-dependent Ca^{2+} leakage (Figure 2), and hence ES24 inhibited the N-glycosylation of Ii to a similar extent as ES1 and ES2, while ES47 had no effect (Figures 4C and 4D).

In summary, ES1 and ES2 efficiently inhibited both protein poly-ubiquitination *in cellula* and ER protein translocation *in vitro*, while ESR35 and ES47 showed no effect on either the ubiquitination or translocation assays (Figure 4). This suggests that ES1 and ES2 have at least two cellular targets within the protein homeostatic network. Firstly, the inhibition of protein translocation into the ER by ES1 and ES2 is most likely due to a direct effect on the Sec61 complex (Cross et al., 2009a). Secondly, ES1 and ES2 may influence de-ubiquitinating processes by interacting directly with p97 (Wang et al., 2008; McKibbin et al., 2012). This dichotomy between Sec61- and p97-mediated effects is apparently absent in the cellular action of ES24, which efficiently inhibits ER translocation but has little if any effect on protein de-ubiquitination (Figure 4). Thus, our results suggest that ES24 selectively inhibits protein translocation at the ER, most likely by interacting with Sec61 complexes. This view is strongly supported by the enhancement of Sec61-dependent Ca^{2+} leakage that is observed when cells are treated with either ES1 or ES24.

Differential Effects of ES Compounds on Cell Death

ES1 has been shown to induce cell death in human blood cancer cell lines, and the 5-NF domain of ES1 was apparently pivotal to this cytotoxic effects (Wang et al., 2010; Auner et al., 2013). To evaluate the role of ER Ca^{2+} leakage in the cytotoxicity of ES compounds, we next compared the sensitivities of various cell lines with ES1, ES24, and ESR35 (Figure 5). HEK, HeLa, INS-1, and NALM-6 cells were first exposed to these compounds for 24 h at concentrations ranging between 0.1 and 20 μM, and cell viability was then evaluated using the Guava ViaCount assay. As shown in Figure 5A, ES1 was toxic to all cell lines tested. As predicted from the lack of effect of ESR35 on Ca^{2+} flux and protein processing (Figures 2 and 4), ESR35 was not toxic to the cell lines tested in the present study (Figure 5A). ES24 exhibited a surprisingly mild cytotoxicity on INS-1 and NALM-6 cells, and had no effect on the viability of HEK and HeLa cells at concentrations up to 20 μM (Figure 5A). Based on the well-defined effects of ES24 on Ca^{2+} leakage and protein translocation (Figures 2 and 4) we hypothesized that, in contrast to the more complex effects of ES1 (Wang et al., 2010), the cytotoxicity of ES24 may primarily reflect its effects on Sec61 complexes. To correlate the toxic effects of ES1 and ES24 with an enhancement of ER Ca^{2+} leakage, we determined the cell tolerance to the disruption of Ca^{2+} homeostasis by treatment with TG for 24 h. As with ES24, we found that HEK and HeLa cells were insensitive to TG (cell viability: HEK, 117.90% ± 3.32%; HeLa, 106.28% ± 2.90%), while INS-1 and NALM-6 cells were more prone to the effects of disruption of the Ca^{2+} homeostasis by TG (cell viability: INS-1, 81.46% ± 5.18%; NALM-6, 75.06% ± 5.20%). Combining the cell viability

data for ES24 and TG, we observed a striking correlation between cell viabilities in the presence of ES24 and TG (Figure 5B), indicating that cell lines with high sensitivity to TG are also more sensitive to ES24. The effects of ES1 and ESR35 on cell viability were not correlated with the cell viabilities under TG treatment (not shown). Although our cytotoxicity studies did not address any potential contribution of the inhibition of protein translocation at ER, our data highlight the role of Sec61-mediated Ca^{2+} leakage in the cytotoxic effects of ES24 (Figure 5). Since TG prevents Ca^{2+} influx into the ER by inhibiting SERCA pumps, the TG effect can be seen as the counterpart of an enhancement of Ca^{2+} leakage. Therefore, the linear relationship between the cytotoxic effects of ES24 and TG shown in Figure 5B suggests that the actions of TG and ES24 may mechanistically converge on ER Ca^{2+} depletion. Conversely, the cytotoxic effects of ES1 may be dominated by p97 inhibition.

DISCUSSION

Growing evidence indicates that Sec61 complexes become permeable for Ca^{2+} ions in the wake of protein translocation and generate a leakage of Ca^{2+} from ER (Lang et al., 2017). Chemical modulators that inhibit protein translocation at the level of Sec61 complexes may, therefore, have an impact on ER Ca^{2+} leakage. Combining biochemical and functional assays, we show two diametrically opposed effects of ES1 on Sec61 complexes. ES1 inhibits protein translocation *in vitro* and enhances Sec61-mediated Ca^{2+} leakage *in cellula*. To gain insight into the mechanism behind the action of ES1 on Sec61 complexes, we compared various ES1 analogs and found that the 5-NF moiety of ES1 is critically involved in the effects on Sec61 complexes. Accordingly, ES24, which closely resembles the 5-NF moiety of ES1, displayed the strongest effects on Ca^{2+} leakage and the cytotoxic effects of ES24 were strongly Ca^{2+} dependent.

ES1 has been show to inhibit protein degradation at the level of p97, an effect that requires the 5-NF domain (Wang et al., 2010). In this study, we observed an accumulation of poly-ubiquitinated species consistent with such an inhibition of protein degradation by ES1 (Figure 4). It is unclear whether these effects of ES1 on protein degradation disturb Ca^{2+} homeostasis in the ER, but, given that ES24 has a strong effect on Ca^{2+} leakage without a comparable effect on ubiquitin homeostasis, these processes may be distinct. Nevertheless, it is conceivable that the cellular accumulation of poly-ubiquitinated proteins over a period of hours may affect Ca^{2+} leakage. For instance, an increase in cytosolic Ca^{2+} levels is detected hours after exposure to proteasome inhibitors (Williams et al., 2013). Notably, in the present study, we found that that ES1 enhanced ER Ca^{2+} leakage in a timescale of seconds to minutes (Figures 1, 2, and 3). Furthermore, silencing of *SEC61A1* abolished the ES1 effects on ER Ca^{2+} leakage completely (Figure 3), indicating that Sec61 complexes are central targets for the action of ES1 at the ER. Like ES1, ES2 and ES24 both enhance ER Ca^{2+} leakage (Figure 2) and inhibit protein translocation (Figures 4C and 4D; see also Cross et al., 2009a), and we assume that these effects also reflect the interaction of ES2 and ES24 with Sec61 complexes. Compared with ES1 and ES2, however, ES24 showed the strongest effects on ER Ca^{2+} leakage generating a robust response within seconds (Figure 2). Effects on Sec61-mediated Ca^{2+} leakage with a similar timescale have been described for inducers of ER stress and protein synthesis inhibitors (Lomax et al., 2002; Van Coppenolle et al., 2004; Lang et al., 2011a; Schäuble et al., 2012). Remarkably, a 10-min exposure to ES1 is also sufficient to observe a reduction in Sec61-mediated protein translocation, suggesting the ES1-mediated inhibition of this process is also a rapid event (Cross et al., 2009a).

The dual effects on Sec61 complexes of ES1, ES2, and ES24 (Figures 3 and 4) indicate that active ES compounds prevent protein translocation into the ER while maintaining Sec61 complexes in a Ca^{2+}-permeable state. Thus, the question arises as to how these apparently opposite effects on protein translocation and Ca^{2+} leakage can be reconciled in a mechanistic model for the action of ES compounds on Sec61 complexes. Using the crystal structure of canine Sec61α (Voorhees and Hegde, 2016), we modeled the structure of Sec61α in the open state (Figure 6A). According to the current view, Sec61α is formed by ten transmembrane helices arranged in two halves (Rapoport et al., 2017). In the idle state, Sec61α is closed by the pore ring and the plug structure. After priming by the ribosome binding, the transmembrane helices rotate in a way that the lateral gate formed by transmembrane helices 2 and 7 is opened and the aqueous channel within Sec61α becomes free for protein translocation (Voorhees and Hegde, 2016). Docking analysis with the Sec61α structure revealed that ES1 interacts with Sec61α with a high affinity ($\Delta G = -9.62$ kcal/mol). Specifically, the 5-NF group of ES1 interacts with H7 and H8 and occupies the volume between H2 and H7 (Figure 6B). Our model predicts that ES24 also binds with a high affinity ($\Delta G = -9.04$ kcal/mol) and interacts with the N-terminal region of H7 (Figure 6C). Remarkably, the aromatic ring of ES24 and the 5-NF group of ES1 docked at the same place surrounded by H2, H7, and H8 at the cytosolic end of the lateral gate. Docking the 5-NF domain alone revealed that it also binds preferentially at the cytosolic end of the lateral gate (Figure S4). This putative interaction site differs from the presumptive binding sites of other Sec61 inhibitors that have been proposed to stabilize the plug domain in a closed state and to prevent allosterically the opening of Sec61 complexes (van Puyenbroeck and Vermeire, 2018). We have estimated the distance of these presumptive binding sites to the lateral gate and found that, with the exception of T86M, they are quite distant and, therefore, unlikely to affect the binding of ES compounds (Figure S5A). Docking analysis with a structural model of the T86M mutant predicted that T86M has only minor effects on the docking of ES1 and ES24 (Figure S5B). The interactions of ES1 and ES24 with Sec61α are predicted to adopt rather central positions at short distances to the center of the H2-H7 gap (9.98 and 9.87 Å for ES1 and ES24, respectively). By contrast, the closest distance between ESR35 and the H2-H7 gap was calculated to be 15.06 Å, and the estimated affinity of its interaction with ESR35 is lower ($\Delta G = -7.91$ kcal/mol). We do not know whether the interaction of ES1 and ES24 with Sec61α is reversible, but a parsimonious model suggests that these ES compounds interact with the helices forming the lateral gate and hamper conformational changes of Sec61α. This is equivalent to proposing that ES1 and ES24 promote an open, Ca^{2+}-permeable, state of Sec61α by preventing the closing of the lateral gate (Figure 6D). Compared with other ion channels, this mechanism

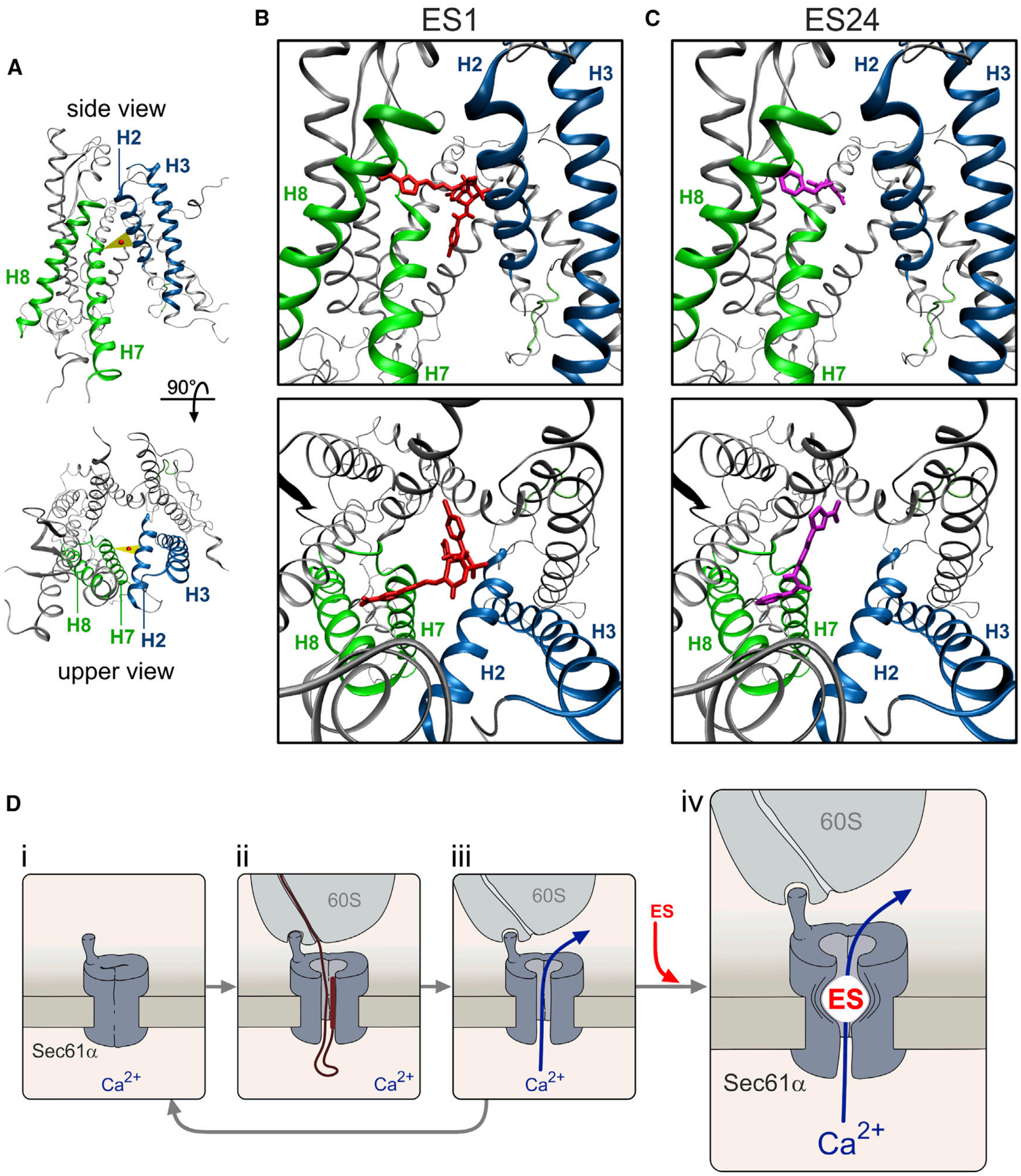

Figure 6. Molecular Modeling of the Interactions of ES1 and ES24 with Sec61α

(A) Homology model of the human Sec61α with the lateral gate in the open conformation. Lateral gate helices are highlighted in colors (H2 and H3, blue; H7 and H8, green). The triangle (yellow) shows the center of mass for the opening between H2 and H7. Overviews depict the Sec61α model seen from the plane of the membrane with the cytosolic side upward (upper panel, lateral gate in front) and from the cytosol (lower panel, cytosolic side in front).

(B and C) Detailed pictures of the docking positions of ES1 (B) and ES24 (C) with the highest scores (ES1, $\Delta G = -9.62$ kcal/mol; ES24, $\Delta G = -9.04$ kcal/mol) in side views (upper panels) and top views (upper panels).

(D) The proposed mode of action of ES1 and ES24 compounds (ES) on Sec61 complexes consider the binding of these compounds to Sec61α in the open state. Idle Sec61 complexes (i) are engaged in co-translational translocation processes upon binding of ribosome nascent chain complexes. Subsequently, the lateral gate of Sec61 complexes opens and the protein-conducting channel allows the translocation of the nascent chain (ii). After translocation, Sec61 complexes might adopt a Ca^{2+}-permeable state with an aqueous channel that allows Ca^{2+} leakage from ER (iii). The cycle is closed when the ribosomes de-attach and Sec61 complexes return to the idle state. By binding to Sec61 complexes in the open state, ES compounds prevent the closing of the lateral gate with the consequence that Sec61 complexes are progressively captured in a Ca^{2+}-permeable state (iv). For simplicity, only the channel-forming subunit (Sec61α) and the large ribosome subunit (60S) are depicted.

See also Figures S4 and S5.

is quite unique in that ES compounds prevent closing and leave the Sec61α channel open for ion permeation. For instance, earlier studies on the action of small molecules on voltage-dependent Na^+ channels have identified a foot in the door effect, in which the compound enters the open channel and prevents the gate from closing by binding within the pore (e.g., Yeh and Armstrong, 1978). In general, however, the consequence of such a foot in the door effect is that ion movements through the pore are blocked. Our model implies that ES compounds keep the lateral gate open as a foot in the door allowing Ca^{2+} ions to move freely through the aqueous channel present in Sec61α after protein translocation (Figure 6D).

ES1 has been proposed as an anticancer agent (van Puyenbroeck and Vermeire, 2018) and, accordingly, aqueous soluble derivatives of ES1 have been developed for cancer therapy (Ding et al., 2016). As shown previously with multiple myeloma cell lines (Auner et al., 2013), we found that the cytotoxicity of ES1 varied between cell lines. ES1 and ES24 not only enhanced the Sec61-mediated Ca^{2+} leakage from ER (Figures 2 and 3), but also inhibited protein translocation (Figure 4). Our model predicts that these ES compounds enhance the Ca^{2+} leakage in the short term and, in the long term, deplete the pool of translocation-competent Sec61 complexes and thereby restrict the process of co-translational protein translocation (Figure 6). We found that the gain of Ca^{2+} leakage induced by ES24 was the predominant mechanism in the cytotoxicity of the compound and that the degree of toxicity depends on cell type (Figure 5). Thus, our study identified ES24 as a small molecule that targets Sec61 complexes and causes cell-type-specific Ca^{2+}-dependent cell death.

SIGNIFICANCE

Small molecules that target Sec61 complexes have been described as antimicrobial and anticancer agents (van Puyenbroeck and Vermeire, 2018). While inhibition of protein translocation by these compounds has been a focus of research, their effects on the ion channel properties of Sec61 complexes are poorly understood. Sec61 complexes can support Ca^{2+} leakage from ER (Lang et al., 2017), hence modulation of Sec61 gating may affect Ca^{2+} homeostasis in the cell. Here we show that inhibition of protein translocation by ES1 is associated with a gain of Sec61-mediated Ca^{2+} leakage from the ER lumen. Collectively, our data suggest that the 5-NF ring of ES1 slides into and obstructs the lateral gate, capturing Sec61 complexes in a Ca^{2+}-permeable, open state. This unprecedented mechanism of action best resembles a "foot in the door" effect, in which ion channel closing is prevented without interference of the ion-conducting pathway. We have tested selected ES1 analogs and found that ES24, in which the chemical structure is basically reduced to the 5-NF domain of ES1, has higher selectivity toward Sec61 complexes than the parental ES1. Hence, among the ES1 analogs tested, ES24 showed the strongest effects on Sec61-mediated Ca^{2+} leakage and, consequently, the cytotoxic effects of ES24 were Ca^{2+} dependent. Thus, our work illustrates a strategy with therapeutic potential, namely one which targets cellular Ca^{2+} homeostasis by taking advantage of the ion channel properties of Sec61 complexes.

STAR★METHODS

Detailed methods are provided in the online version of this paper and include the following:

- KEY RESOURCES TABLE
- CONTACT FOR REAGENT AND RESOURCES SHARING
- EXPERIMENTAL MODEL AND SUBJECT DETAILS
- METHOD DETAILS
  - Synthesis of Eeyarestatin (ES) Compounds
  - *In Vitro* Translocation Assay
  - *In Cellula* Ubiquitination Assay
  - Gene Silencing
  - Live Cell Calcium Imaging
  - Cell Viability Assay
  - Homology Modelling and Docking Protocols
- QUANTIFICATION AND STATISTICAL ANALYSIS
- DATA AND SOFTWARE AVAILABILITY

SUPPLEMENTAL INFORMATION

Supplemental Information includes five figures and can be found with this article online at https://doi.org/10.1016/j.chembiol.2019.01.010.

ACKOWLEDGEMENTS

We thank Roger Y. Tsien, Bernhard Dobberstein, and Per-Olof Berggren for kindly providing us with the calcium sensor D1ER, the HLA class II histocompatibility antigen gamma chain, and the INS-1 cell line, respectively. We acknowledge Dr. Veit Flockerzi (Saarland University) for helpful comments, Dr. Stephan Philipp (Saarland University) for his help in generating the HEK D1ER cell line, and Heidi Löhr and Martin Simon-Thomas (Saarland University) for their excellent technical assistance. This work was supported by the Deutsche Forschungsgemeinschaft (DFG) grants SFB 894(to R.Z. and A.C.) and IRTG 1830 (to R.Z.), the Biotechnology and Biological Sciences Research Council (BBSRC) Doctoral Training Program Award BB/J014478/1 (S.O.), and the Welcome Trust Investigator Award in Science (2049)57/Z/16/Z (to S.H.).

AUTHOR CONTRIBUTIONS

I.G., S.O'K., T.P., M.-C.K., D.N., and C.M. performed the experiments and analyzed the data. V.H., E.S., S.H., R.Z., and A.C. supervised the experiments. M.P., H.M.W., S.L. F., R.C.W., and E.S. devised, developed, and synthesized the ES compounds. S.H., R.Z., and A.C. designed the study and wrote the manuscript with the contribution of all authors.

DECLARATION OF INTERESTS

The authors declare no competing interests.

Received: May 30, 2018
Revised: October 6, 2018
Accepted: January 24, 2019
Published: February 21, 2019

REFERENCES

Aletrari, M.O., McKibbin, C., Williams, H., Pawar, V., Pietroni, P., Lord, J.M., Flitsch, S.L., Whitehead, R., Swanton, E., High, S., and Spooner, R.A. (2011). Eeyarestatin 1 interferes with both retrograde and anterograde intracellular trafficking pathways. PLoS One *6*, e22713.

Aller, S.G., Yu, J., Ward, A., Weng, Y., Chittaboina, S., Zhuo, R., Harrell, P.M., Trinh, Y.T., Zhang, Q., Urbatsch, I.L., and Chang, G. (2009). Structure of P-glycoprotein reveals a molecular basis for poly-specific drug binding. Science *323*, 1718–1722.

Asfari, M., Janjic, D., Meda, P., Li, G., Halban, P.A., and Wollheim, C.B. (1992). Establishment of 2-mercaptoethanol-dependent differentiated insulin-secreting cell lines. Endocrinology *130*, 167–178.

Auner, H.W., Moody, A.M., Ward, T.H., Kraus, M., Milan, E., May, P., Chaidos, A., Driessen, C., Cenci, S., Dazzi, F., et al. (2013). Combined inhibition of p97 and the proteasome causes lethal disruption of the secretory apparatus in multiple myeloma cells. PLoS One *8*, e74415.

Camello, C., Lomax, R., Petersen, O.H., and Tepikin, A.V. (2002). Calcium leak from intracellular stores – the enigma of calcium signalling. Cell Calcium *32*, 355–361.

Carreras-Sureda, A., Pihán, P., and Hetz, C. (2018). Calcium signaling at the endoplasmic reticulum: fine-tuning stress responses. Cell Calcium *70*, 24–31.

Claesson, L., Larhammar, D., Rask, L., and Peterson, P.A. (1983). cDNA clone for the human invariant gamma chain of class II histocompatibility antigens and its implications for the protein structure. Proc. Natl. Acad. Sci. U S A *80*, 7395–7399.

Cross, B.C.S., McKibbin, C., Callan, A.C., Roboti, P., Piacenti, M., Rabu, C., Wilson, C.M., Whitehead, R., Flitsch, S.L., Pool, M.R., et al. (2009a). Eeyarestatin I inhibits Sec61-mediated protein translocation at the endoplasmic reticulum. J. Cell Sci. *122*, 4393–4400.

Cross, B.C.S., Sinning, I., Luirink, J., and High, S. (2009b). Delivering proteins for export from cytosol. Nat. Rev. Mol. Cell Biol. *10*, 255–264.

Ding, R., Zhang, T., Xie, J., Williams, J., Ye, Y., and Chen, L. (2016). Eeyarestatin I derivatives with improved aqueous solubility. Bioorg. Med. Chem. Lett. *26*, 5177–5181.

Dudek, J., Pfeffer, S., Lee, P.H., Jung, M., Cavalié, A., Helms, V., Förster, F., and Zimmermann, R. (2015). Protein transport into the human endoplasmic reticulum. J. Mol. Biol. *427*, 1159–1175.

Erdmann, F., Schäuble, N., Lang, S., Jung, M., Honigmann, A., Ahmad, M., Dudek, J., Benedix, J., Harsman, A., Kopp, A., et al. (2011). Interaction of calmodulin with Sec61a limits Ca^{2+} leakage from the endoplasmic reticulum. EMBO J. *30*, 17–31.

Fiebiger, E., Hirsch, C., Vyas, J.M., Gordon, E., Ploegh, H.L., and Tortorella, D. (2004). Dissection of the dislocation pathway for type I membrane proteins with a new small molecule inhibitor, eeyarestatin. Mol. Biol. Cell *15*, 1635–1646.

Figueiredo Costa, B., Cassella, P., Colombo, S.F., and Borgese, N. (2018). Discrimination between endoplasmic reticulum and mitochondria by spontaneously inserting tail-anchored proteins. Traffic *19*, 182–197.

Lang, S., Erdmann, F., Jung, M., Wagner, R., Cavalié, A., and Zimmermann, R. (2011a). Sec61 complexes form ubiquitous ER Ca^{2+} leak channels. Channels *5*, 228–235.

Lang, S., Schäuble, N., Cavalié, A., and Zimmermann, R. (2011b). Live cell calcium imaging combined with siRNA mediated gene silencing identifies Ca^{2+} leak channels in the ER membrane and their regulatory mechanisms. J. Vis. Exp. *53*, e2730.

Lang, S., Pfeffer, S., Lee, P.-H., Cavalié, A., Helms, V., Förster, F., and Zimmermann, R. (2017). An update on Sec61 channel functions, mechanisms and related diseases. Front. Physiol. *8*, 887.

Le Gall, S., Neuhof, A., and Rapoport, T.A. (2004). The endoplasmic reticulum membrane is permeable to small molecules. Mol. Biol. *15*, 447–455.

Lomax, R.B., Camello, C., Van Coppenolle, F., and Petersen, O.H. (2002). Basal and physiological Ca^{2+} leak from the endoplasmic reticulum of pancreatic acinar cells. Second messenger-activated channels and translocons. J. Biol. Chem. *277*, 26479–26485.

Martí-Renom, M.A., Stuart, A.C., Fiser, A., Sánchez, R., Melo, F., and Sali, A. (2000). Comparative protein structure modeling of genes and genomes. Annu. Rev. Biophys. Biomol. Struct. *29*, 291–325.

McKibbin, C., Mares, A., Piacenti, M., Williams, H., Roboti, P., Puumalainen, M., Callan, A.C., Lesiak-Mieczkowska, K., Linder, S., Harant, H., et al. (2012). Inhibition of protein translocation at the endoplasmic reticulum promotes activation of the unfolded protein response. Biochem. J. *442*, 639–648.

Means, S., Smith, A.J., Shepherd, J., Shadid, J., Fowler, J., Wojcikiewicz, R.J.H., Mazel, T., Smith, G.D., and Wilson, B.S. (2006). Reaction diffusion model of calcium dynamics with realistic ER geometry. Biophys. J. *91*, 537–557.

Morris, G.M., Huey, R., Lindstrom, W., Sanner, M.F., Belew, R.K., Goodsell, D.S., and Olson, A.J. (2009). AutoDock4 and AutoDockTools4: automated docking with selective receptor flexibility. J. Comput. Chem. *30*, 2785–2791.

Naydenov, N.G., Harris, G., Brown, B., Schaefer, K.L., Das, S.K., Fischer, P.B., and Ivanov, A.I. (2012). Loss of soluble *N*-ethylmaleimide-sensitive factor attachment protein α(αSNAP) induces epithelial cell apoptosis via down-regulation of Bcl-2 expression and disruption of the golgi. J. Biol. Chem. *297*, 5928–5941.

Palmer, A.E., Jin, C., Reed, J.C., and Tsien, R.Y. (2004). Bcl-2-mediated alterations in endoplasmic reticulum Ca^{2+} analysed with an improved genetically encoded fluorescent sensor. Proc. Natl. Acad. Sci. U S A *101*, 17404–17409.

Pfeffer, S., Burbaum, L., Unverdorben, P., Pech, M., Chen, Y., Zimmermann, R., Beckmann, R., and Forster, F. (2015). Structure of the native Sec61 protein-conducting channel. Nat. Commun. *6*, 8403.

Phillips, J.C., Braun, R., Wang, W., Gumbart, J., Tajkhorshid, E., Villa, E., Chipot, C., Skeel, R.D., Kalé, L., and Schulten, K. (2005). Scalable molecular dynamics with NAMD. J. Comput. Chem. *26*, 1781–1802.

Rapoport, T.A., Li, L., and Park, E. (2017). Structural and mechanistic insight into protein translocation. Annu. Rev. Cell Dev. Biol. *33*, 369–390.

Rasmussen, S.G., Choi, H.J., Fung, J.J., Pardon, E., Casarosa, P., Chae, P.S., Devree, B.T., Rosenbaum, D.M., Thian, F.S., Kobilka, T.S., et al. (2011). Structure of a nanobody-stabilized active state of the b2 adrenoceptor. Nature *469*, 175–180.

Schäuble, N., Cavalié, A., Zimmermann, R., and Jung, M. (2014). Interaction of *Pseudomonas aeruginosa* Exotoxin A with the human Sec61 complex suppresses passive calcium efflux from the endoplasmic reticulum. Channels *8*, 76–83.

Schäuble, N., Lang, S., Jung, M., Cappel, S., Schorr, S., Ulucan, Ö., Linxweiler, J., Dudek, J., Blum, R., Helms, V., et al. (2012). BiP-mediated closing of the Sec61 channel limits Ca^{2+} leakage from the ER. EMBO J. *31*, 3282–3296.

Simon, S.M., and Blobel, G. (1991). A protein conducting channel in the endoplasmic reticulum. Cell *65*, 371–380.

Sztretye, M., Yi, J., Figueroa, L., Zhou, J., Royer, L., and Eduardo Ríos, E. (2011). D4cpv-calsequestrin: a sensitive ratiometric biosensor accurately targeted to the calcium store of skeletal muscle. J. Gen. Physiol. *138*, 211–229.

Van Coppenolle, F., Vanden Abeele, F., Slomianny, C., Flourakis, M., Hesketh, J., Dewailly, E., and Prevarskaya, N. (2004). Ribosome translocon complex mediates calcium leakage from endoplasmic reticulum stores. J. Cell Sci. *117*, 4135–4142.

van Puyenbroeck, V., and Vermeire, K. (2018). Inhibitors of protein translocation across membranes of the secretory pathway: novel antimicrobial and anticancer agents. Cell. Mol. Life Sci. *75*, 1541–1558.

Voorhees, R.M., and Hegde, R.S. (2016). Structure of the Sec61 channel opened by a signal sequence. Science *351*, 88–91.

Wang, Q., Li, L., and Ye, Y. (2008). Inhibition of p97-dependent protein degradation by Eeyarestatin I. J. Biol. Chem. *283*, 7445–7454.

Wang, Q., Mora-Jensen, H., Weniger, M.A., Perez-Galan, P., Wolford, C., Hai, T., Ron, D., Chen, W., Trenkle, W., Wiestner, A., and Ye, Y. (2009). ERAD inhibitors integrate ER stress with an epigenetic mechanism to

activate BH3-only protein NOXA in cancer cells. Proc. Natl. Acad. Sci. U S A *106*, 2200–2205.

Wang, Q., Shinkre, B.A., Lee, J.G., Weniger, M.A., Liu, Y., Chen, W., Wiestner, A., Trenkle, W.C., and Ye, Y. (2010). The ERAD inhibitor eeyarestatin I is a bifunctional compound with a membrane-binding domain and a p97/VCP inhibitory group. PLoS One *5*, e15479.

Williams, J.A., Hou, Y., Ni, H.-M., and Ding, W.-X. (2013). Role of intracellular calcium in proteasome inhibitor-induced endoplasmic reticulum stress, autophagy and cell death. Pharm. Res. *30*, 2279–2298.

Wiederstein, M., and Sippl, M.J. (2007). ProSA-web: interactive web service for the recognition of errors in three-dimensional structures of proteins. Nucleic Acids Res. *35*, W407–W410.

Wirth, A., Jung, M., Bies, G., Frien, M., Tyedmers, J., Zimmermann, R., and Wagner, R. (2003). The Sec61p complex is a dynamic precursor activated channel. Mol. Cell *12*, 261–268.

Yeh, J.Z., and Armstrong, C.M. (1978). Immobilisation of gating charge by a substance that stimulates inactivation. Nature *273*, 387–389.

STAR★METHODS

KEY RESOURCES TABLE

REAGENT or RESOURCE	SOURCE	IDENTIFIER
**Antibodies**		
Anti-Sec61 antibody (against the carboxy terminal peptide (14-mer) of human Sec61α)	This study	N/A
Anti-Serca2 ATPase	Sigma-Aldrich	Cat# S1439; RRID: AB_261442
Polyclonal anti-β-actin antibody<br>Monoclonal anti-β-actin antibody	Abcam<br>Sigma-Aldrich	Cat# Ab8227; RRID: AB_2305186<br>Cat# A5441; RRID: AB_476744
Mono- and polyubuitinylated conjugates monoclonal antibody (FK2)	Enzo Life Sciences	Cat# PW8810; RRID: AB_10541840
IRDye 680 RD Goat anti-Rabbit IgG (H + L) Secondary Antibody	LI-COR Biotechnology	Cat# 926-68071; RRID: AB_10956166
IRDye 800 RD Goat anti-Mouse IgG (H + L) Secondary antibody	LI-COR Biotechnology	Cat# 926-32210; RRID: AB_621842
**Chemicals, Peptides, and Recombinant Proteins**		
Eeyarestatin 1 (ES1)	This study	N/A
Eeyarestatin 2 (ES2)	This study	N/A
ES24	This study	N/A
ESR35	This study	N/A
ES47	This study	N/A
Thapsigargin	ThermoFisher Scientific	T7459
Ionomycin	ThermoFisher Scientific	I24222
Nigericin	Sigma-Aldrich	N7143
2-APB	Tocris	524-95-8
(-)-Xestospongin C	Tocris	88903-69-9
FURA-2 AM	ThermoFisher Scientific	F1221
Casein Blocking Buffer 10x	Sigma-Aldrich	B6429
Puromycin dihyrochloride from Streptomyces *alboniger*	Sigma-Aldrich	P7255
Minimal Essential Medium (MEM)	ThermoFidher Scientific	31095-029
Dulbecco's Modified Eagle's Medium (DMEM)	ThermoFisher Scientific	41966-029
RPMI 1640	ThermoFisher Scientific	21875-034
G418	Sigma-Aldrich	G8168
Foetal bovine serum (FBS)	ThermoFisher Scientific	11573397
L-alanyl-L-Glutamine	ThermoFisher Scientific	35050-038
β-Mercaptoethanol	Carl Roth	4227.3
Sodium-Pyruvate	ThermoFisher Scientific	11360-070
Amino Acid Mixture Minus Methionine	Promega	L996A
Reticulocyte Lysate, Nuclease Treated	Promega	L416A
EasyTag EXPRESS^{35}S Protein Labeling Mix, [^{35}S]	PerkinElmer	NEG7720002MC
HiPerFect Transfection Reagent	Qiagen	301705
Guava ViaCount	Merck	4000-0041
**Deposited Data**		
Human Sec61 subunit alpha, isoform 1	Dudek et al., 2015	UniProtKB: P61619
Canine Sec61 alpha 1 structure	Voorhees and Hegde, 2016	PDB: 3JC2
Canine ribosome-bound Sec61 alpha 1 structure	Pfeffer et al., 2015	PDB: 5A6U

(*Continued on next page*)

Continued

REAGENT or RESOURCE	SOURCE	IDENTIFIER
Mouse P-glycoprotein structure	Aller et al., 2009	PDB: 3G5U
Human beta 2 adrenoreceptor structure	Rasmussen et al., 2011	PDB; 3P0G
Experimental Models: Cell Lines		
HEK	ATCC	CRL-1573
HEK-D1ER	This study	N/A
HeLa	ATCC	CCL-2
INS-1	Asfari et al., 1992	N/A
NALM-6	DSMZ	ACC-128
Oligonucleotides		
SEC61A1#4 siRNA: GGAAUUUGCCUGCUAAUCAtt	Qiagen	N/A
SEC61A1#5 siRNA: CACUGAAAUGUCUACGUUUtt	Qiagen	N/A
AllStars Negative Control siRNA	Qiagen	1027285
SERCA2 siRNA	ThermoFisher Scientific	120744
Silencer Negative Control	ThermoFisher Scientific	AM4611
Recombinant DNA		
pcDNA3-D1ER	Palmer et al., 2004	Addgene: 36325
cDNA coding for residues 17 to 232 of the short form of human HLA class II histocompatibility antigen gamma chain (Ii)	Claesson et al., 1983	UniProtKB: P04232-2
Software and Algorithms		
Live Acquisition	Till Photonics	https://www.fei.com/service-support/Light-Microscopy/
AIDA v5.0	Elysia Raytest	http://www.elysia-raytest.com/de/cataloglight/
Matlab R2015b	Mathworks	https://de.mathworks.com/products.html
MODELLER 9.17	Martí-Renom et al., 2000	https://salilab.org/modeller/download_installation.html
NAMD	Phillips et al., 2005	http://www.ks.uiuc.edu/Development/Download/download.cgi?PackageName=NAMD
Pro-Sa-web	Wiederstein and Sippl, 2007	https://prosa.services.came.sbg.ac.at/prosa.php
AutoDock4	Morris et al., 2009	http://autodock.scripps.edu/downloads

CONTACT FOR REAGENT AND RESOURCES SHARING

Further information and requests for resources and reagents should be directed to and will be fulfilled by the Lead Contact, Adolfo Cavalié (Adolfo.cavalie@uks.eu).

EXPERIMENTAL MODEL AND SUBJECT DETAILS

HEK cells (human epithelial embryonic kidney cells, ATCC) were cultivated in Dulbecco's Modified Eagle's Medium (DMEM) supplemented with 10% (v/v) foetal bovine serum (FBS). In order to obtain cells with homogenous expression levels of the FRET-based Ca^{2+}-sensor D1ER, we generated the stable cell line HEK D1ER by transfecting HEK cells with the plasmid pcDNA3-D1ER (Palmer et al., 2004), which was kindly provided R. Y. Tsien (University of California San Diego, La Jolla, USA). HEK-D1ER cells were maintained in culture under selection with G418 (0.5 mg/ml) in Minimal Essential Medium (MEM) supplemented with 10% (v/v) FBS. HeLa cells (human epithelial cervix carcinoma cells, ATCC, female) were cultured in DMEM supplemented with 10% (v/v) FBS and 2 mM *L*-alanyl-*L*-glutamine. NALM-6 (human B-cell precursor leukaemia cells, DSMZ, male) were cultured in RPMI 1640 medium supplemented with 10% (v/v) heat-inactivated FBS. INS-1 cells are derived from rat insulinoma (Asfari et al., 1992) and were

kindly provided by P. O. Berggren (Karolinska Institute, Stockholm, Sweden). The culture medium of INS-1 cells was RPMI 1640 supplemented with 10% (v/v) heat-inactivated FBS, 50 μM β-mercaptoethanol and 1 mM Na-pyruvate. All cell lines were maintained in a 5% CO_2 humidified incubator at 37°C.

METHOD DETAILS

Synthesis of Eeyarestatin (ES) Compounds

The compounds ES1, ES2 and ESR35 were synthesized as previously described (McKibbin et al., 2012).

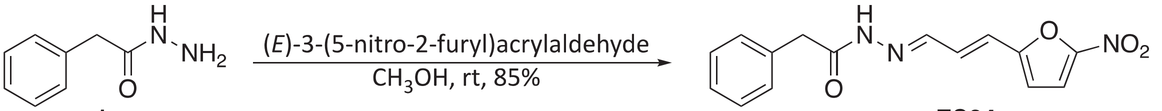

1 **ES24**

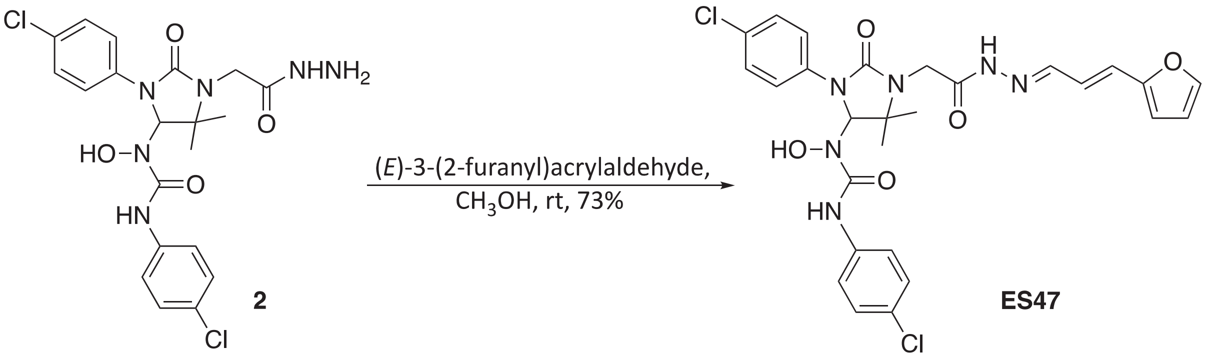

2 **ES47**

ES24 and ES47 were prepared in good yields by condensation of acyl hydrazides **1** and **2** with the appropriate conjugated aldehyde. Phenylacetic hydrazide **1** is commercially available and the synthesis of **2** (ESR35) has been reported previously (Cross et al., 2009a). The double bond geometry in both compounds was confirmed by the magnitude of the corresponding vicinal ^{1}H-^{1}H coupling constant (15.9 Hz for ES24 and 14.8 Hz for ES47).

General experimental synthetic reagents and solvents were purchased from Sigma-Aldrich or Alfa Aesar and used as supplied. IR spectra were recorded on a Perkin Elmer 881 spectrometer, an AT1-Matson Genesis Series FTIR spectrometer or a Perkin Elmer Spectrum BX FTIR spectrometer. ^{1}H NMR spectra were recorded on a Bruker Avance II 500MHz spectrometer. Chemical shifts are referenced to the residual solvent peak. Mass spectra were recorded on a Micromass Platform II (electrospray) spectrometer. Melting points were recorded using a Sanyo Gallenkamp MPD350 heater and are uncorrected.

2-{2-[3-(5-nitro-2-furyl)-2-propen-1-ylidene]hydrazino}-2-oxoethylbenzene (ES24)

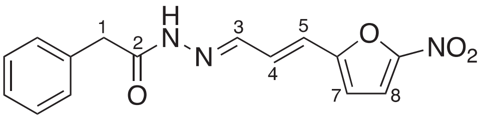

(*E*)-3-(5-Nitro-2-furyl)acrylaldehyde (128 mg, 0.77 mmol) was added, under nitrogen, to a solution of phenylacetic hydrazide (115 mg, 0.77 mmol) in anhydrous CH_3OH (4 mL). The reaction mixture was stirred at room temperature overnight and then Et_2O was added to allow complete precipitation to occur. The solid was collected by filtration and washed with Et_2O and pentane, yielding the title compound (196 mg, 85%) as a yellow solid. Mp 202-204°C; ν_{max} (evaporated film)/cm^{-1} 3137 (w, N-H), 3040 (w, C-H), 2971 (brw, C-H), 2857 (brw, C-H), 1747 (w, C=O), 1667 (s, C=C), 1587 (m, C=C), 1557 (m, C=C), 1504 (s, C=C); ^{1}H NMR ($CDCl_3$; 500 MHz) δ 4.03 (2H, s, C(1)H_2), 6.63 (1H, br, C(7)H), 6.65 (1H, d, *J* = 15.9 Hz, C(5)H), 7.15 (1H, dd, *J* =15.9, 9.5 Hz, C(4)H), 7.33-7.37 (6H, m, C(8) H & Ar-CH), 7.51 (1H, d, *J* = 9.5 Hz, C(3)H); *m/z* (-ve ion electrospray) 298 ([M-H]$^-$, 100%), (+ve ion electrospray) 322 ([M+Na]$^+$, 100%); found 322.0805, $C_{15}H_{13}N_3O_4Na$ ([M+Na]$^+$), requires 322.0798.

N'-(4-Chlorophenyl)-N-[3-(4-chlorophenyl)-2-oxo-1-(2-{2-[3-(2-furyl)-2-propen-1-ylidene]hydrazino}-2-oxoethyl)-5,5-dimethyl-4-imidazolidinyl]-N-hydroxyurea (ES47)

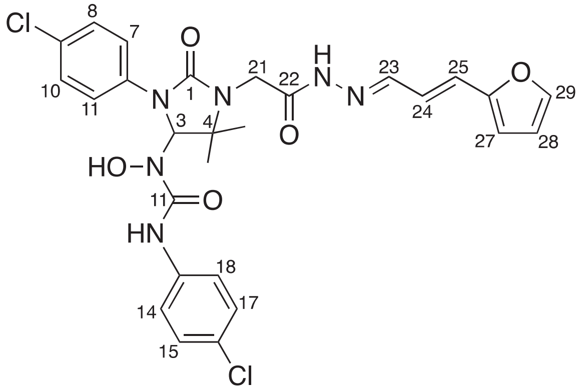

(*E*)-3-(2-Furanyl)acrylaldehyde (49 mg, 0.4 mmol) was added, under nitrogen, to a solution of **2** (96 mg, 0.2 mmol) in anhydrous CH_3OH (1 mL). The reaction mixture was stirred at room temperature for 2 days and then concentrated under reduced pressure. The residue was recrystallised at room temperature from Et_2O /pentane, yielding the title compound (86 mg, 73%) as a pale yellow solid. Mp 213-214.5°C; ν_{max} (solid state)/cm^{-1} 3395 (w, O-H), 3222 (brw, N-H), 3145 (brw, N-H), 3132 (brw), 3039 (w, C-H), 2979 (w, C- H), 2974 (w, C-H), 2930 (w, C-H), 1699 (m, C=O), 1680 (s, C=O), 1661 (s, C=O), 1633 (m, C=C), 1588 (w, C=C), 1550 (s, C=C), 1512 (m, C=C); ^{1}H NMR ($CDCl_3$; 500 MHz) δ 1.31 (3H, s, C(19)H_3 or C(20)H_3), 1.40 (3H, s, C(19)H_3 or C(20)H_3), 4.41 (2H, ABq, J_{AB} = 18.7 Hz, C(21)H_2), 5.75 (1H, d, *J* = 14.8 Hz, C(25)H), 6.10 (1H, s, C(3)H), 6.29 (1H, d, *J* = 3.1 Hz, C(27)H), 6.38 (1H, dd, *J* = 3.1, 1.7 Hz, C(28)H), 6.59 (1H, dd, *J* = 14.8, 7.9 Hz, C(24)H), 7.17 (4H, m, Ar-CH), 7.20-7.25 (1H, m, C(23)H), 7.40 (1H, br, C(29)H), 7.47 (4H, m, Ar-CH), 7.78 (1H, brs, NH or OH), 9.41 (1H, brs, NH or OH), 9.75 (1H, brs, NH or OH); *m/z* (−ve ion electrospray) 587 ($[M-H]^-$, (^{37}Cl,^{37}Cl), 10%), 585 ($[M-H]^-$, (^{35}Cl,^{37}Cl), 65%), 583 ($[M-H]^-$, (^{35}Cl,^{35}Cl), 100%); (+ve ion electrospray) 611 ($[M+Na]^+$, (^{37}Cl, ^{37}Cl), 10%) 609 ($[M+Na]^+$, (^{35}Cl,^{37}Cl), 60%), 607 ($[M+Na]^+$, (^{35}Cl,^{35}Cl), 100%); (Found 607.1244, $C_{27}H_{26}{}^{35}Cl_2N_6NaO_5$, ($[M+Na]^+$), requires 607.1234).

In Vitro Translocation Assay

Nuclease-treated canine pancreatic microsomes were treated with 250 μM ESR35, ES1, ES2, ES24, ES47 (from 1 mM stock solutions in DMSO), or an equal volume of DMSO, and incubated on ice for 1 h before inclusion in translocation analyses. Cell-free translation (20 μL) was performed in nuclease-treated rabbit reticulocyte lysate (Promega) in the presence of EasyTag EXPRESS ^{35}S Protein Labelling Mix containing [^{35}S] methionine (Perkin Elmer) (0.611 MBq; 40.95 TBq/mmol), ~2.5% (v/v) amino acids minus methionine (Promega), 9% (v/v) *in vitro* transcribed mRNA (290 ng/μL stock) encoding residues 17 to 232 of the short form of human HLA class II histocompatibility antigen gamma chain (Ii) and 10% (v/v) inhibitor/DMSO pre-treated rough microsomes for 20 min at 30°C. The short form of Ii was kindly provided by Dr. B. Dobberstein (ZMBH, Heidelberg University, Heidelberg, Germany). Following translation, puromycin was added to 0.1 mM and samples incubated for 5 min at 30°C to ensure ribosomal release. The membrane-associated fraction of the reaction was isolated by centrifugation (100,000 *g* for 10 min at 4°C in a TLA100 rotor (Beckmann) through an 80 μL high-salt cushion (0.75 M sucrose, 0.5 M KOAc, 5 mM $Mg(OAc)_2$, 50 mM Hepes-KOH, pH 7.9) and the membrane pellet suspended in 30 μL SDS sample buffer (0.02% bromophenol blue, 62.5 mM, 4% (w/v) SDS, 10% (v/v) glycerol; pH 7.6; 1 M dithio-threitol). Samples were denatured at 70°C for 5 min prior to resolution by SDS-PAGE (10 μL sample, 16% polyacrylamide gels, 120 V, 120 min). Gels were incubated in fix mix (20% MeOH, 10% AcOH) for 5 min, dried (BioRad Gel Dryer) for 2 h at 65°C and exposed to a phosphore imaging plate for 24 h. Radiolabelled products were visualized using a Typhoon FLA-7000 (GE Healthcare). Quantitative analysis was performed with AIDA v5.0 (Raytest Isotopenmeβgeräte) whereby the signal intensity of Ii-2 Gly/Ii was used as a read-out for total translocation and expressed relative to the DMSO control to determine the mean relative ER processing from 3 independent experiments. Data is presented as mean ± SEM.

In Cellula Ubiquitination Assay

Experiments performed whit HeLa cells cultured to ~80% confluent. DMEM was aspirated from each well 16 h after cell plating and cells treated with 8 μM ES1, ES2, ES24, ESR35 and ES47 (from 10 mM stock solutions in DMSO), or an equal volume of DMSO, in 1 mL fresh DMEM for 8 h. Upon treatment completion, DMEM was aspirated, cells washed twice with PBS (Dulbecco's Phosphate Buffered Saline) and solubilized in 100 μL SDS sample buffer. Samples were denatured for 16 h at 37°C, resolved by SDS-PAGE (20 μL sample, 10% polyacrylamide gels, 120 V, 100 min) and proteins transferred to a PVDF membrane in transfer buffer (0.08 M Tris base, 0.60 M glycine, 20% MeOH) at 300 mA for 2.5 h at ambient temperature. The membrane was blocked in Casein blocking buffer (Sigma) diluted in TBS (Tris Buffered Saline; 0.2 M Tris base and 1.5 M NaCl; pH 7.4) for 1 h at ambient temperature prior to primary antibody (FK2 mouse (Enzo Life Sciences) and β-actin (Abcam)) incubation in Casein blocking buffer diluted 1:10 in TBST (TBS supplemented with 0.1% Tween-20) for 16 h at 4°C. After washing with TBST (4 x 10 min), the membrane was then incubated

with a fluorescent secondary antibody for 1 h at ambient temperature. Following washing with TBST (4 x 10 min), samples were visualized using an Odyssey CLx Imaging System (LICOR Biosciences). Ubiquitin signals were normalized relative to the intensity of the β-actin loading control and normalized signals expressed relative to the DMSO solvent control. Data is shown as mean ± SEM from 6 independent experiments.

Gene Silencing

Silencing of *SEC61A1* was performed in HeLa cells as previously described (Lang et al., 2011b). Briefly, 5.2 x 10^5 HeLa cells were seeded per 6-cm culture plate transfected with *SEC61A1*#4 (GGAAUUUGCCUGCUAAUCAtt, Qiagen) or *SEC61A1*#5 siRNA (CACUGAAAUGUCUACGUUUtt, Qiagen) or scramble siRNA (AllStars Negative Control siRNA, Qiagen) using HiPerFect Reagent (Qiagen). After 24 h, cells were transfected for a second time. Ca^{2+} imaging experiments were performed 96 h after beginning of transfection. Silencing efficiencies were evaluated by Western blot analysis using the respective antibodies and an anti-β-actin-antibody from mice (Schäuble et al., 2012). For silencing of SERCA2, HeLa cells were transfected with 30 μM *siRNA SERCA2* (GCGGAUUAAAGCUAAAGACtt, Thermo Fisher) or with 30 μM *scr RNA* (Silencer Negative Control, Thermo Fisher) using HiPerFect (Qiagen). Ca^{2+} imaging experiments were performed 24 h after transfection. *SERCA2* expression levels were detected with a SERCA2 antibody (S1439, Thermo Fisher).

Live Cell Calcium Imaging

$[Ca^{2+}]_{ER}$ and $[Ca^{2+}]_{cyt}$ were imaged using D1ER and FURA-2, respectively. D1ER is a genetically encoded FRET-based Ca^{2+} sensor that comprises two fluorescent proteins, CFP and citrine, and two sensing proteins, a calmodulin-binding peptide derived from skeletal muscle myosin light chain kinase and calmodulin (Palmer et al., 2004). D1ER was stably expressed in HEK cells. FURA-2 is a ratiometric dye that localizes to the cytosol (see Lang et al., 2011b). For simultaneous measurements of $[Ca^{2+}]_{ER}$ and $[Ca^{2+}]_{cyt}$, HEK-D1ER cells were loaded with the membrane-permeable FURA-2 AM just before each Ca^{2+} imaging experiment (see Lang et al., 2011b). In order to prevent the Ca^{2+} entry from the extracellular space, all Ca^{2+} imaging experiments were carried out with a Ca^{2+}-free bath solution (140mM NaCl, 5mM KCl, 1mM MgCl2, 0.5 mM EGTA, 10 mM glucose in 10 mM HEPES-KOH, pH 7.35). Accordingly, D1ER and FURA-2 reported changes in $[Ca^{2+}]_{ER}$ and $[Ca^{2+}]_{cyt}$, respectively, which arose from Ca^{2+} mobilisation within internal cell compartments. Ca^{2+} imaging experiments were performed with cells that were plated on poly-L-lysine-coated cover slips and cultured for 48-72 h.

Ca^{2+} imaging experiments were carried out with the iMIC microscope, the polychromator V, and the Live Acquisition software (Till Photonics). To allow the recording of D1ER and FURA-2 signals in the same cells, the filter sets were automatically exchanged. Firstly, D1ER was excited by exposing HEK D1ER cells at 433 nm. The emitted florescence was then split at 469/23 nm and 536/27 nm to obtain the CFP and citrine components, respectively. The cell fluorescence was additionally passed through a dichrotome and projected on the chip of the microscope camera to obtain simultaneous CFP and citrine images. Secondly, FURA-2 was excited by alternated excitation at 340 and 380nm. The emitted fluorescence also passed through a dichrotome and captured at 510 nm to obtain FURA-2 images at 340 and 380 nm excitation. D1ER and FURA-2 image pairs containing 5–10 cells/frame were obtained every 10-12 s. D1ER ratios were calculated from CFP and citrine image pairs as F536/F469, where F536 and F469 represent the background-subtracted citrine and CFP fluorescence intensities, respectively. FURA-2 signals were measured in FURA-2 image pairs as F340/F380, where F340 and F380 correspond to the background-subtracted fluorescence intensity at 340 and 380 nm excitation wavelengths, respectively. Following corrections for bleed-through, F536/F469 and F340/F380 were used to calculate $[Ca^{2+}]_{ER}$ and $[Ca^{2+}]_{cyt}$, respectively (see Sztretye et al., 2011; Lang et al., 2011b). Basically, free Ca^{2+} concentrations were calculated with the standard ratiometric equation $[Ca^{2+}]_{free} = \beta K_d \cdot ((R-R_{min})/(R_{max}-R))$ (see Lang et al., 2011b), in which we replaced R by the ratios F536/F469 and F340/F380 to obtain $[Ca^{2+}]_{ER}$ and $[Ca^{2+}]_{cyt}$, respectively. In this equation, the apparent K_d of the Ca^{2+} sensors is represented by βK_d and was obtained by measuring F536/F469 ratios in HEK-D1ER cells that were exposed to 10 μM ionomycin, 10 μM nigericin and free Ca^{2+} concentrations between 1 μM and 10 mM. Fitting of the calibration data with the standard ratiometric equation reveal a βK_d of 165.25 μM for D1ER in our imaging system. $\beta K_d \cdot$ was 5.26 μM for FURA-2 in our imaging system. Maximal and minimal fluorescence ratios of the Ca^{2+} sensors are given by R_{min} and R_{max} in the standard ratiometric equation, respectively, HEK-D1ER cells were exposed to 10 μM ionomycin and the R_{max} of D1ER signals was measured as the maximal F536/F469 ratio in the presence of 25 mM Ca^{2+}. Conversely, the R_{min} of D1ER signals was the minimal F536/F469 ratio measured in the presence of 0.5 mM EGTA. The R_{max} and R_{min} of FURA-2 signals were determined in FURA-2 loaded HEK-D1ER cells as previously described (see Lang et al., 2011b). HeLa cells were used to image only cytosolic Ca^{2+} in gene silencing experiments and, accordingly, $[Ca^{2+}]_{cyt}$ was imaged with FURA-2 as previously described (see Lang et al., 2011b).

Stock solution of ES compounds were prepared by dissolving the compounds ES1, ES2, ES24, ESR35 and ES457 in DMSO to obtain concentrations of 10-100 mM. Stock were maintained frozen at -20°C. Similarly, 1 mM stocks of thapsigargin were prepared in DMSO. Just before experiments, stocks of TG and ES compounds were diluted to a 2X concentration in the bath solution, i.e., 2 times greater than the desired final concentration. Routinely, TG and ES compounds were applied to the cells during Ca^{2+} imaging by adding 2X solutions to the bath at a ratio of 1:1 to avoid problems arising from slow mixing. The final DMSO concentration in bath was 0.01-0.05 % v/v. During Ca^{2+} imaging, a base line was routinely recorded before applying ES compounds in order to determine the status of $[Ca^{2+}]_{cyt}$ and $[Ca^{2+}]_{ER}$. In the so-called "online" protocol, ES compounds were applied at 3 min while imaging was running. Mock treatment represents the application of 0.01–0.05% v/v DMSO in bath solution. Following 1, 4 and 17 min exposures to eeyarestatin compounds, TG was applied in bath to unmask the Ca^{2+} leakage from ER. In experiments with D1ER and FURA-2,

5-10 HEK D1ER cells were imaged simultaneously, while 30-50 HeLa cells were imaged per experiment with FURA-2. Time courses of $[Ca^{2+}]_{ER}$ and/or $[Ca^{2+}]_{cyt}$ were constructed for each experiment and parameters such as the area under the curve and amplitude of TG responses were measured for each single cell. Ca^{2+} imaging experiments were performed with 3-5 different batches of HeLa and HEK D1ER cells. Data has been collected in 6-12 independently experiments repeated for each experimental setting and is shown as mean ± SEM.

Cell Viability Assay

HEK, HeLa, INS-1 and NALM-6 cells were plated in culture dishes at densities between $0.25\text{-}0.50 \cdot 10^6$ cells/ml in the respective culture medium and treated with ES1, ES24 and ESR35 at concentrations between 1-20 μM for 24 h. Similarly, cells were treated with 1 μM thapsigargin for 24 h. The DMSO concentration was 0.1 % in TG-containing medium and below 0.02 % in medium with ES compounds. Following treatment, cells were incubated with PBS and Guava ViaCount reagent (1:10) for 5 min in the dark at room temperature. Cell viability was measured by flow cytometry with Guava easyCyte 8HT (Merck Millipore). Viability measurements were performed as duplicates in 3 independent experiments per treatment with 5000 cells/measurement. The cell viability of cells exposed to ES1, ES24 and ESr35 was normalised with respect to the viability of non-treated cells. For cells exposed to TG, cell viability was normalised to 0.1 % DMSO controls. Normalised viability data is presented as mean ± SD.

Homology Modelling and Docking Protocols

The protein sequence of human Sec61α isoform 1, which contains 476 amino acids, was retrieved from the UniProtKB database (UniProt: P61619). The crystal structure of canine Sec61α (PDB: 3JC2) was selected for homology modelling of human Sec61α. Both sequences share 99.8% sequence identity. The 3JC2 structure shows Sec61α in an open conformation but is lacking structural information for the helical plug region (residue 63 to 69). Since in the open conformation, the plug is believed to be outside of the translocation pathway, we assumed that it does not form relevant interaction with the substrates. Homology modelling was carried out using the MODELLER 9.17 package. The plug region was modelled and optimised as a loop structure. Subsequently, the homology model was subjected to energy minimization, using the NAMD package, to relax side chain atoms. The resulting model (Figure 6A) was validated by the ProSa-web server, along with two Sec61α structures (PDB: 3JC2 and PDB: 5A6U) and two other membrane protein structures: a GPCR (PCB: 3P0G) and an ABC transporter (PDB: 3G5U). The analysis from ProSa-web showed that the score of the homology model is within the range of scores typically found in experimentally determined structures of protein chains. The structural model of Sec61 α carrying the T86M mutation was generated by Swiss-Pdb Viewer and subsequent minimization with NAMD.

The protonation states of protein residues and partial charges of ligands were assigned by the *prepare_receptor* and *prepare_ligand* modules of the AutoDock4 package. For each ligand, the docking calculations were performed in 2 consecutive steps: In the first docking step, we adopted a relatively large grid box covering the entire cavity of Sec61, to scan for energetically favourable conformations of the ligand inside the Sec61 pocket. The Lamarckian genetic algorithm was used for the optimization of the ligand conformations and orientations (cubic box of 45 Å, grid spacing, 2.5×10^6 energy evaluations and 27 $\times10^3$ generations). In the second docking step, the size of the grid box was scaled down based on the population of the most stable binding positions of the ligand. In a finer run, more stringent parameters (cubic box of 34 Å, 100×10^6 energy evaluations and 0.5×10^6 generations) were used.

QUANTIFICATION AND STATISTICAL ANALYSIS

Quantification procedures used *in vitro* and *in cellula* experiments as well as in Ca^{2+} imaging experiments are described in Method Details.

The statistical significance of *in vitro* and *in cellula* data with respect to DMSO controls was determined using Tukey's and Dunnett's multiple comparison tests, respectively. Statistical significance of the Ca^{2+} imaging data was assessed with the two-sample Kolmogorov-Smirnov test. Statistical significance is given as n.s., non-significant; *, $P < 0.05$; **, $P < 0.01$ and ***, $P < 0.001$.

DATA AND SOFTWARE AVAILABILITY

In vitro data was analysed with AIDA. Matlab was used in the analysis of Ca^{2+} imaging data. Homology modelling and docking analysis were performed with Modeller 9.17, NAMD, Pro-Sa-web and AutoDock4.

Cell Chemical Biology, Volume *26*

Supplemental Information

Eeyarestatin Compounds Selectively Enhance Sec61-Mediated Ca^{2+} Leakage from the Endoplasmic Reticulum

Igor Gamayun, Sarah O'Keefe, Tillman Pick, Marie-Christine Klein, Duy Nguyen, Craig McKibbin, Michela Piacenti, Helen M. Williams, Sabine L. Flitsch, Roger C. Whitehead, Eileithyia Swanton, Volkhard Helms, Stephen High, Richard Zimmermann, and Adolfo Cavalié

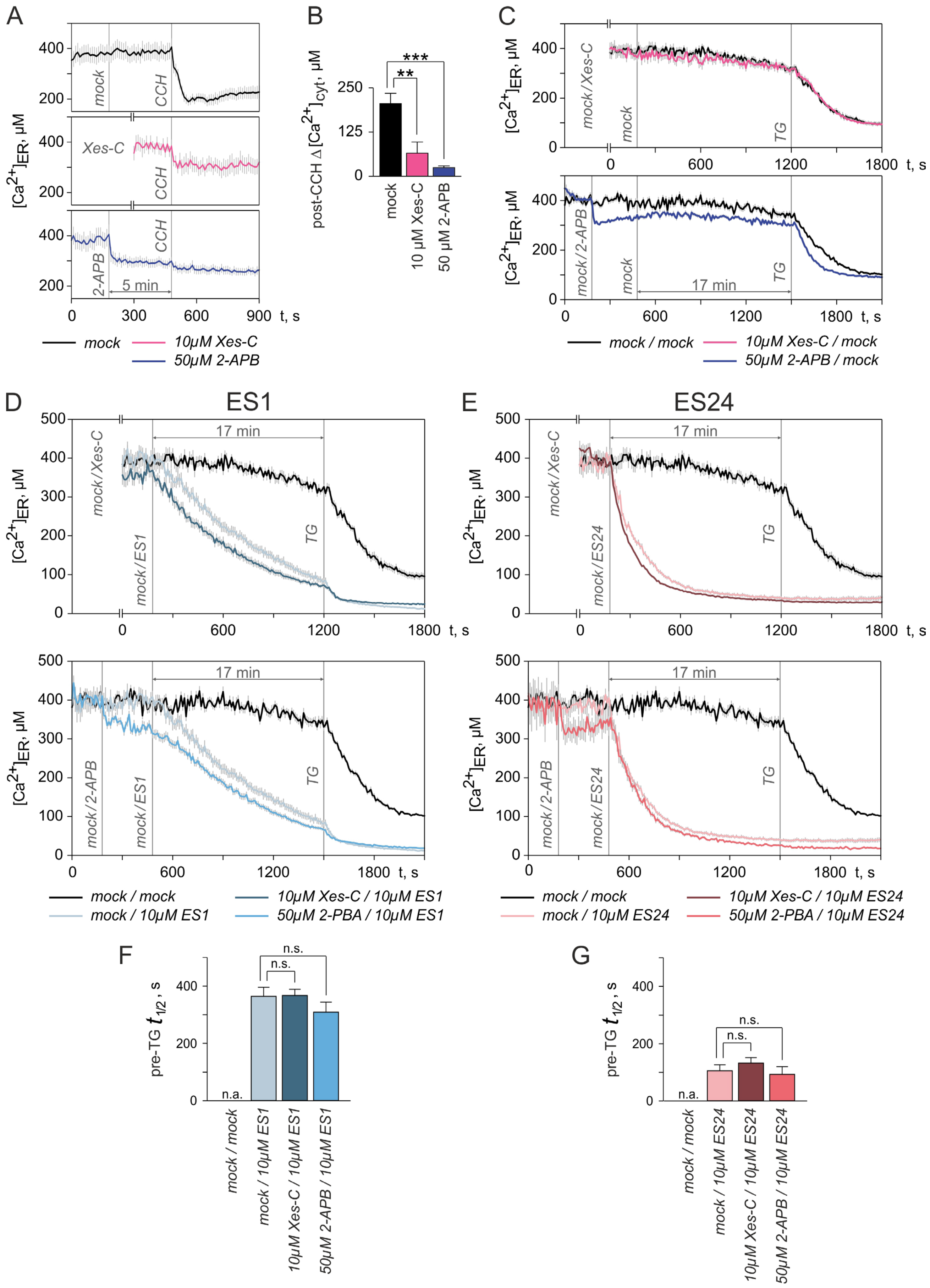

FIGURE S1 (related to Fig. 1)

FIGURE S1 (related to Fig. 1). Effects of ES1 and ES24 on the Ca^{2+} homeostasis under inhibition of IP_3 receptors.

(**A**) Assessing the inhibition of IP_3 receptors with 2-aminoethoxydiphenyl borate (2-APB) and xestospongin C (Xes-C) in HEK D1ER cells. The Ca^{2+} concentration in ER ($[Ca^{2+}]_{ER}$) was measured in the absence of extracellular Ca^{2+} (see Methods) and the functionality of the IP_3 signalling pathway was assessed by applying 1 mM carbachol (CCH). In mock-treated cells, CCH induced a drop in ER Ca^{2+}, indicating that the IP_3 signalling pathway is active in HEK D1ER cells (upper panel). The ER Ca^{2+} drop induced by CCH was strongly reduced in cells pre-treated with 10 µM Xes-C for 20 min (middle panel) and almost abolished after exposure to 50 µM 2-APB (lower panel).
(**B**) The quantification of decreases in $[Ca^{2+}]_{ER}$ induced by CCH (post-CCH $\Delta[Ca^{2+}]_{ER}$) indicates that 50 µM 2-APB inhibits IP_3 receptors stronger than 10 µM Xes-C in HEK D1ER cells.
(**C**) As a control, the protocol used in the experiments shown in Fig. 1B and Fig. 2C was tested in cells treated with Xes-C (10 µM Xes-C / mock) and 2-APB (50 µM 2-APB / mock). The time courses of $[Ca^{2+}]_{ER}$ in non-treated cells (mock / mock) and in cells treated with Xes-C were similar (upper panel). Although 2-APB induced a drop in ER Ca^{2+}, $[Ca^{2+}]_{ER}$ remained constant during the recordings (lower panel). As in Fig.1B and 2C, 1 µM thapsigargin (TG) was applied at the end of recordings.
(**D** – **E**) Effects of 10 µM ES1 and 10 µM ES24 on $[Ca^{2+}]_{ER}$ under inhibition of IP_3 receptors with Xes-C and 2-APB. HEK D1ER cells were pre-treated with 10 µM Xes-C for 20 min and, subsequently, ES1 and ES24 were applied (*upper panels*, 10 µM Xes-C / 10 µM ES1, 10 µM Xes-C / 10 µM ES24). The exposure to 2-APB was 5 min long and followed by application of ES1and ES24 (*lower panels*, 50 µM 2-APB / 10 µM ES1, 50 µM 2-APB / 10 µM ES24). For comparison, time courses of $[Ca^{2+}]_{ER}$ in control experiments (mock / mock) and the effects of ES1 and ES24 on naïve cells (mock / 10 µM ES1; mock / 10 µM ES24) are shown.
(**F** – **G**) Comparison of half-times of the $[Ca^{2+}]_{ER}$ decay induced by ES1 and ES24 before TG application (pre-TG $t_{1/2)}$ in the experiments shown in D-E.

DMSO controls are denoted as mock. Application time points are indicated by vertical lines in A and C-G. Data is presented as mean ± SEM. n.a., not analysed; n.s., non-significant.
**, P < 0.01; ***, P < 0.001. N: 9-15 cells per experimental setting.

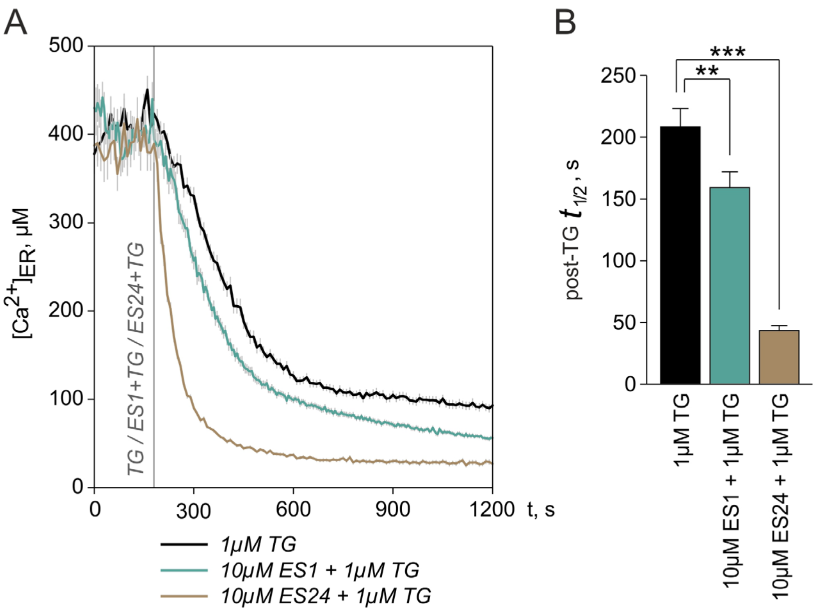

FIGURE S2 (related to Fig. 2). ES1 and ES24 accelerate the ER Ca^{2+} depletion induced by inhibition of SERCA pumps with thapsigargin.

(**A**) The Ca^{2+} concentration in ER ($[Ca^{2+}]_{ER}$) was measured in HEK D1ER cells in the absence of extracellular Ca^{2+} (see Methods) and 1 μM thapsigargin was applied either alone (TG) or in combination with 10 μM ES1 (TG+ES1) and 10 μM ES24 (TG+ES24) as indicated. (**B**) Comparison of the half times of decay in $[Ca^{2+}]_{ER}$ (post-TG $t_{1/2}$) induced by the application of TG, TG+ES1 and TG+ES24 in the experiments shown in A. Data is presented as mean ± SEM. **, $P < 0.01$; ***, $P < 0.001$. N: 10-13 cells per experimental setting.

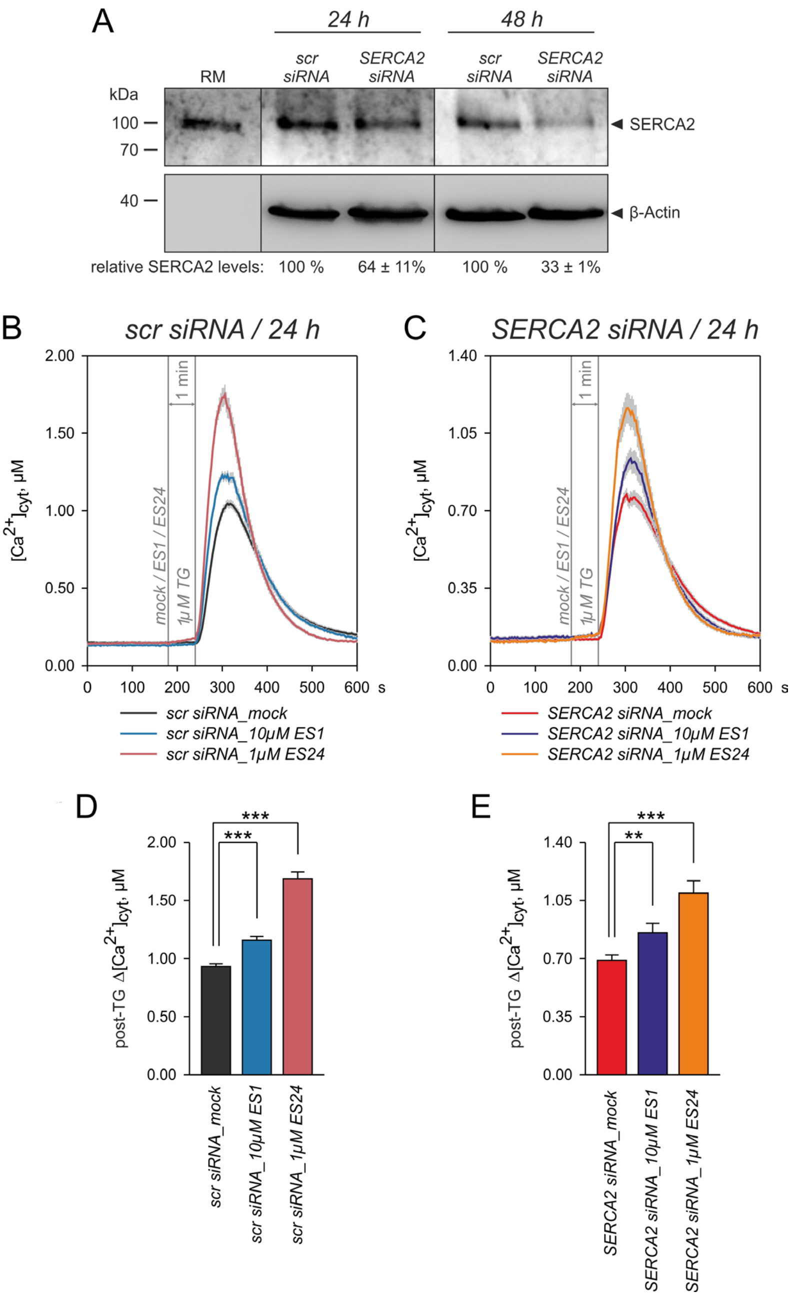

FIGURE S3 (related to Fig. 3). Effects of ES1 and ES24 in HeLa cells after knockdown of SERCA pumps.

(**A**) Expression of *SERCA2* in HeLa cells transfected with scramble siRNA (*scr siRNA*) or with siRNA targeting *SERCA2* (*SERCA2 siRNA*). SERCA2 protein levels were normalized to β-actin and expressed relative to levels in cells transfected with *scr siRNA*. RM, pancreatic rough microsome. Relative SERCA2 protein levels are given below Western blots.
(**B – C**) Effects of 10 µM ES1 and 1 µM ES24 on HeLa cells transfected with *scr siRNA* or *siRNA SERCA2*, as indicated above graphs.
(**D – E**) Quantification of TG responses (post-TG $\Delta[Ca^{2+}]_{cyt}$) measured in experiments shown in B-C.

DMSO controls are denoted as mock. Application time points of ES1, ES24 and TG are indicated by vertical lines in B-C. Data is presented as mean ± SD in A and as mean ± SEM in B-E. N: 3 blots (A) and 48-63 cells per experimental setting (B-H). **, P < 0.01 and ***, P < 0.001.

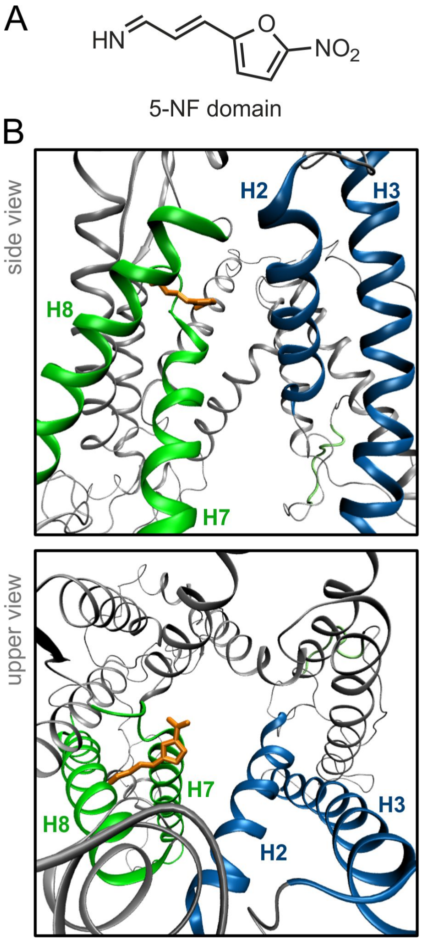

FIGURE S4 (related to Fig. 6). Docking of the 5-nitrofuran (5-NF) domain to Sec61α.

(**A**) Chemical structure of the 5-NF domain.
(**B**) Docking of the pure 5-NF domain was analysed using the homology model of human Sec61α. Shown is the docking position of the 5-NF domain (orange) with best docking score (ΔG = -5.69 kcal/mol). While this is lower than the docking scores of ES1 and ES24, it is typical that larger molecules achieve larger docking scores. The distance of 5-NF to the H2-H7 gap is 8.65 Å and, thus, slightly shorter than for ES1 (9.98 Å, see Fig. 6) and ES24 (9.87 Å, see Fig. 6). Images show the Sec61α model seen from the plane of the membrane with the cytosolic side upwards (upper panel) and from the cytosol (lower panel). Lateral gate helices are highlighted in colours (H2 and H3, blue; H7 and H8, green).

A

Mutation	Minimal distance (Å)
D60G	27.29
M65R	33.99
R66I	37.19
S71F	28.50
G80V	13.15
S82P	7.07
T86M	3.78
Y131H	18.27
M136T	26.43

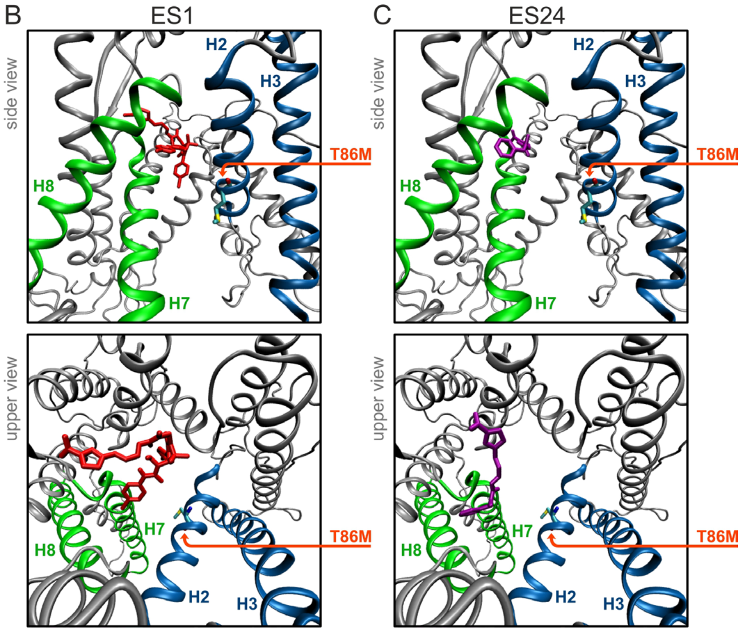

FIGURE S5 (related to Figure 6). Docking positions of ES1 and ES24 in the homology model of the human Sec61α with the mutation T86M.

(**A**) Previously described Sec61α mutations that confer resistance to various translocation inhibitors (Van Puyenbroeck and Vermiere, 2018) were incorporated in the homology model and minimal distances between mutated amino acids (C-alpha atom positions) and the H2-H7 gap of the lateral gate were measured. Among all these mutants, T86M is closest to the lateral gate. Hence, the mutation T86M was incorporated in the homology Sec61α model and docking of ES1 and ES24 to the structural model of this mutant was performed.
(**B** – **C**) Best-scored docking positions of ES1 and ES24 in the model of the T86M Sec61α mutant. The positions of ES1 and ES24 in the T86M Sec61α mutant are very similar to the respective docking positions in the model of native Sec61α (see Fig. 6). The docking scores of ES1 and ES24 in the T86M mutant were slightly lower (ES1, ΔG = -9.39 kcal/mol; ES24, ΔG = -8.57 kcal/mol) than in the native Sec61α (ES1, ΔG = -9.62 kcal/mol; ES24, ΔG = -9.04 kcal/mol; see Fig. 6). Images show side views (upper panels) and upper views (lower panels) of the Sec61α model seen from the plane of the membrane with the cytosolic side upwards and from the cytosol, respectively. The position of T86M is indicated in orange. Lateral gate helices are highlighted in colours (H2 and H3, blue; H7 and H8, green).
